# Structural insights into broadly neutralizing antibodies elicited by hybrid immunity against SARS-CoV-2

**DOI:** 10.1080/22221751.2022.2146538

**Published:** 2023-01-03

**Authors:** Mengxiao Luo, Biao Zhou, Eswar R. Reddem, Bingjie Tang, Bohao Chen, Runhong Zhou, Hang Liu, Lihong Liu, Phinikoula S. Katsamba, Ka-Kit Au, Hiu-On Man, Kelvin Kai-Wang To, Kwok-Yung Yuen, Lawrence Shapiro, Shangyu Dang, David D. Ho, Zhiwei Chen

**Affiliations:** a AIDS Institute, Li Ka Shing Faculty of Medicine, The University of Hong Kong, Pokfulam, Hong Kong Special Administrative Region, People’s Republic of China; b Department of Microbiology, School of Clinical Medicine, Li Ka Shing Faculty of Medicine, The University of Hong Kong, Pokfulam, Hong Kong Special Administrative Region, People’s Republic of China; c Zuckerman Mind Brain Behaviour Institute, New York, NY, USA; d Division of Life Science, Center of Systems Biology and Human Health, The Hong Kong University of Science and Technology, Clear Water Bay, Kowloon, Hong Kong Special Administrative Region, People’s Republic of China; e Aaron Diamond AIDS Research Center, Columbia University Vagelos College of Physicians and Surgeons, New York, NY, USA; f State Key Laboratory of Emerging Infectious Diseases, The University of Hong Kong, Pokfulam, Hong Kong Special Administrative Region, People’s Republic of China; g Centre for Virology, Vaccinology and Therapeutics, Health@InnoHK, The University of Hong Kong, Pokfulam, Hong Kong Special Administrative Region, People’s Republic of China; h Department of Clinical Microbiology and Infection Control, The University of Hong Kong-Shenzhen Hospital, Shenzhen, People’s Republic of China; i Department of Microbiology, Queen Mary Hospital, Pokfulam, Hong Kong Special Administrative Region, People’s Republic of China; j Southern Marine Science and Engineering Guangdong Laboratory (Guangzhou), Guangzhou, People’s Republic of China; k HKUST-Shenzhen Research Institute, Nanshan, People’s Republic of China

**Keywords:** SARS-CoV-2, Omicron BA.1 breakthrough infection, hybrid immunity, broadly neutralizing antibody, memory B cell, structural basis of antibody

## Abstract

Increasing spread by SARS-CoV-2 Omicron variants challenges existing vaccines and broadly reactive neutralizing antibodies (bNAbs) against COVID-19. Here we determine the diversity, potency, breadth and structural insights of bNAbs derived from memory B cells of BNT162b2-vaccinee after homogeneous Omicron BA.1 breakthrough infection. The infection activates diverse memory B cell clonotypes for generating potent class I/II and III bNAbs with new epitopes mapped to the receptor-binding domain (RBD). The top eight bNAbs neutralize wildtype and BA.1 potently but display divergent IgH/IgL sequences and neuralization profiles against other variants of concern (VOCs). Two of them (P2D9 and P3E6) belonging to class III NAbs display comparable potency against BA.4/BA.5, although structural analysis reveals distinct modes of action. P3E6 neutralizes all variants tested through a unique bivalent interaction with two RBDs. Our findings provide new insights into hybrid immunity on BNT162b2-induced diverse memory B cells in response to Omicron breakthrough infection for generating diverse bNAbs with distinct structural basis.

## Introduction

The continuous emergence of severe acute respiratory syndrome coronavirus 2 (SARS-CoV-2) variants of concern (VOCs) has posed challenges for COVID-19 vaccine efficacy and pandemic control [1,2]. In early November 2021, the Omicron variant BA.1 (B.1.1.529) was discovered in Botswana and South Africa and became dominated quickly by replacing VOC Delta worldwide [3]. Thereafter, several Omicron sub-lineages including BA.2, BA.2.12.1, BA.2.13, BA.3, BA.4 and BA.5 have been discovered [4]. BA.2 and its sub-lineages have increased rapidly in several countries or regions such as South Africa, Denmark, Sweden, India, Singapore and Hong Kong [5,6]. BA.2.12.1 has accounted for more than 50% of COVID-19 cases in the United States at the end of May 2022 (https://covid.cdc.gov/covid-data-tracker). Recently, BA.4 and BA.5 have become dominant in South Africa since January 2022 with trends to outcompete BA.2 and BA.2.12.1. At the end of June, BA.4 and BA.5 made up more than half of new COVID-19 cases in partial India, Singapore, the UK and the United States [4]. To date, BA.1-derived vaccines have been developed as boosters with the hope to elicit broad-spectrum protection against newly emerging Omicron variants [7–9], yet the types of human neutralizing antibodies (NAbs) generated during natural BA.1 breakthrough infections among vaccinees remain incompletely understood.

One critical feature that distinguishes Omicron sub-lineages from previous VOCs is the increased number of mutations accommodated by the receptor-binding domain (RBD), leading to possible antigenic shifts and antibody evasion [10]. BA.1 has 15 RBD mutations whereas BA.1.1 contains an extra R346K mutation [11]. BA.2 does not contain G446S and G496S but has four different mutations including T376A, D405N, R408S and S371F compared with BA.1 [11]. The sub-lineage BA.2.12.1 contains more mutations such as L452Q and S704L whereas BA.2.13 has L452M. The recent BA.4 and BA.5 variants evolve further with L452R and F486V [9]. These two mutations confer even stronger evasion to both vaccine-induced neutralizing antibody responses and neutralization activities of many class I/II and class III NAbs [9]. Since it is now essential to investigate broadly-reactive NAbs (bNAbs) for protection against emerging Omicron variants [12–14], we conducted an in-depth study on the diversity, potency and breadth of NAbs generated by hybrid immunity in fully-vaccinated individual after BA.1 natural infection.

## Results

### Broadly reactive antibody responses recalled by Omicron BA.1 breakthrough infection

**Figure 1.**
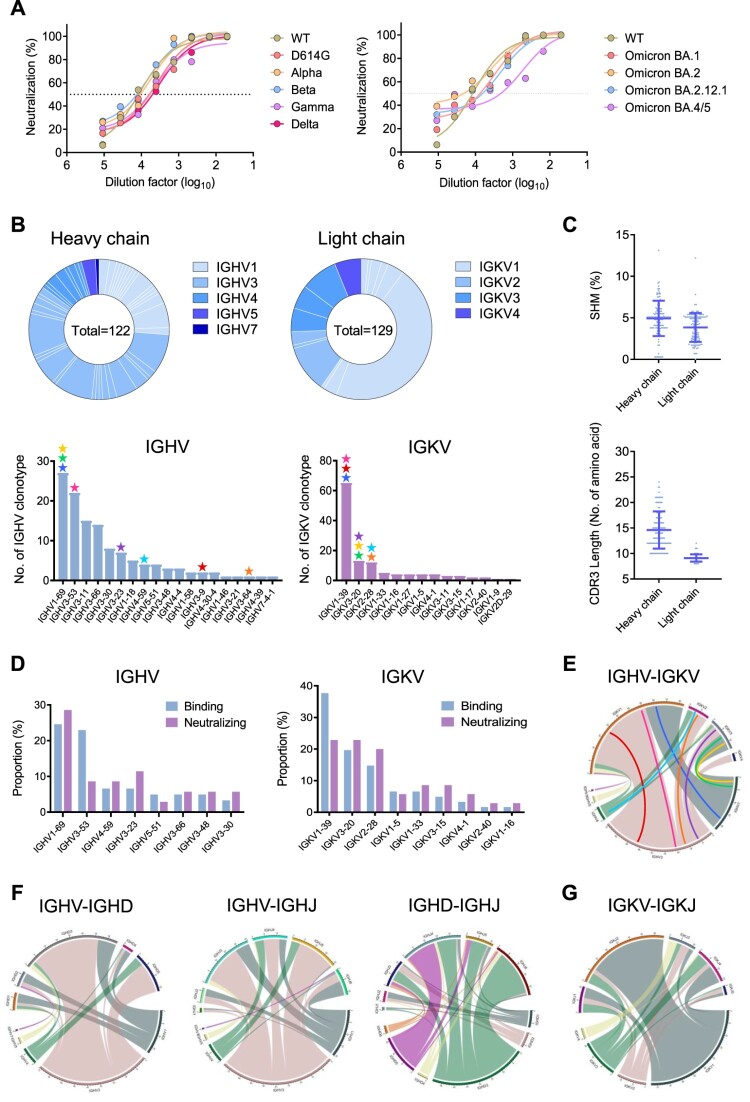
Omicron BA.1 breakthrough infection recalled broadly reactive antibody responses and activated diverse memory B cell clonotypes. (A) The neutralization activity of plasma derived from the patient Omicron-01 was determined against pseudotyped SARS-CoV-2 WT and variants including Omicron sub-lineages. (B) Antibody gene repertoire analysis of reactive memory B cells derived from Omicron-01. In each pie chart, the number of cloned antibody genes is shown in the centre for the heavy (H) or light (L) chains. The colours represent variable gene families and each fragment of the same colour stands for one specific sub-family. The histograms summarize the number of antibodies encoded by each IGHV or IGKV gene sub-family. The coloured stars indicate the IG genes of eight identified antibodies: P1D9 (blue), P1F8 (orange), P2B4 (red), P2B11 (green), P2D9 (yellow), P2E7 (cyan), P3E2 (magenta) and P3E6 (purple). (C) The percentage of somatic hypermutation (SHM) compared to germline sequences and the CDR3 amino acid lengths of recovered H and L chain sequences are presented in scatter dot plots. (D) The gene enrichment of 63 mAbs displaying SARS-CoV-2 WT spike-binding activity is compared to that of 35 mAbs with neutralization activity. The proportions of IGHV and IGKV genes encoding those antibodies are shown in the bar. (E) The chord chats display the variable pairings between recovered H chain and L chain V genes. The outer circle border indicates the number of each pairing. The inner-coloured lines represent P1D9 (blue), P1F8 (orange), P2B4 (red), P2B11 (green), P2D9 (yellow), P2E7 (cyan), P3E2 (magenta) and P3E6 (purple), respectively. (F) The chord charts display the variable combination of V, D and J genes of 122 recovered H chains. The outer circle border indicates the number of each combination. (G) The chord chart displays the variable combination of V and J genes of 129 recovered L chains. The outer circle border indicates the number of each combination.

Our first local case of SARS-CoV-2 Omicron BA.1 breakthrough infection was found in the quarantine hotel in Hong Kong in November 2021 [15]. Before the infection, this subject (Omiron-01) had received two doses of mRNA vaccines (BNT-162b2) around 180 days before symptom onset. At the time of symptom onset, the subject had nearly unmeasurable NAb activities against the WT virus [14]. Viral sequencing analysis revealed that the breakthrough infection was caused by a homogenous BA.1 strain [15], which has subsequently been used for the development of inactivated vaccines in China. The subject developed an increased amount of cross-reactive Abs to wildtype (WT), Beta (B.1.351), Gamma (P1), Delta (B.1.617.2) and Omicron BA.1 (B.1.1.529) 9 days after the infection compared with the other vaccinee with breakthrough infection [14]. In this study, we further determined the plasma neutralizing activity at the time of B cell sorting against a broad spectrum of SARS-CoV-2 VOCs including newly emerged Omicron variants. We found that the plasma displayed the mean EC_50_ at 1:5887 dilution for binding to BA.1 spike, only relatively weaker than to D614G (Figure S1A), indicating potential generation of BA.1-reactive antibody responses. Moreover, the plasma sample exhibited potent neutralizing capability against not only SARS-CoV-2 VOCs Beta (B.1.351), Gamma (P1), Delta (B.1.617.2) and Omicron BA.1 (B.1.1.529) but also recently emerged Omicron sub-lineages BA.2, BA.2.12.1 and BA.4/5, with the mean IC_50_ at 1:7135 dilution (Figure 1(A, B)). The IC_50_ value to BA.4/5, however, was 1:525.8, showing 7.4- and 6-fold drop compared with D614G and BA.1, respectively (Figure S1B). These results indicated that the homogenous BA.1 breakthrough infection induced cross-reactive polyclonal NAb responses against diverse SARS-CoV-2 VOCs and multiple Omicron sub-lineages.

### Omicron BA.1 breakthrough infection activated diverse memory B cell clonotypes for generating bNAbs

To delineate the memory B cell repertoire activated by the Omicron BA.1 breakthrough infection, antigen-specific immunoglobulin G-positive (IgG^+^) memory B cells were sorted from PBMCs at the single cell level using SARS-CoV-2 WT/Alpha/Beta/Gamma/Delta RBD and spike extracellular domain (S-ECD) as the bait (Figure S2). The S-ECD positive memory B cells were about 4.46% of total memory B cells, which was higher than previous reports by us and others [16,17]. A total of 161 S-ECD reactive memory B cells were obtained, resulting in 122 heavy chain and 129 light chain sequences successfully recovered after the sequencing confirmation (Figure 1(B), top). Based on the antibody repertoire analysis, we found that multiple B cell clonotypes were activated in the heavy chain including IGHV1-69 (22.1%), IGHV3-53 (18.0%), IGHV3-11 (12.3%) and IGHV3-66 (11.5%), whereas IGKV1-39 (50.4%) was the most predominant light chain (Figure 1(B), bottom). Notably, the involvement of IGHV3-53, IGHV3-66 and IGHV1-69 has been recently documented for antibodies isolated from other BA.1 breakthrough infection cases [18,19]. Moreover, both heavy chain and light chain had low rates of somatic hypermutation (SHM) with the mean values of 4.9% and 3.8%, respectively (Figure 1(C), top), and short complementarity determining region 3 (CDR3) with the mean length of 14.6 and 9.1, respectively (Figure 1(C), bottom), in line with previous findings [20,21]. These results demonstrated that the homogenous Omicron BA.1 breakthrough infection was able to activate a pool of memory B cell clonotypes as polyclonal antibody responses.

From the recovered antibody sequences of individual B cells, we successfully expressed 104 monoclonal antibodies (mAbs) with naturally paired V_H_ and V_L,_ and 60.6% (63/104) of them were able to bind to SARS-CoV-2 WT spike as determined by ELISA (OD450 > 0.1; Figure S3A). Compared to the antibody repertoire, these 63 mAbs were predominantly encoded by IGHV1-69 (24.6%) and IGHV3-53 (23.0%) for heavy chains, whereas the light chain was mainly IGKV1-39 (37.7%) (Figure 1(D)). Using the pseudovirus neutralization assay, we revealed that 55.6% (35/63) of these spike-specific mAbs showed neutralization activity, mainly targeting RBD (Figure S3C and Table S1). Notably, 48.6% (17/35) of these NAbs were able to cross-neutralize both WT and Omicron BA.1 (Figure S3B). The remaining 37.1% (13/35) and 14.3% (5/35) NAbs neutralized WT and Omicron BA.1, respectively. For these NAbs, although IGHV1-69 was more frequently used, the IGVK gene was not restricted to 1–39 (Figure 1(D)). The pairing of heavy chain and light chain, therefore, did not focus on public B cell clonotypes such as IGHV3-53/3-66/IGKV1-39 and IGHV1-58/IGKV3-20 (Figure 1(E)). Instead, the V-D-J recombination for heavy chain and the V-J recombination for light chain were diverse with IGHV3-IGHD3/IGHD5 and IGHV3-IGHJ3/IGHJ5 for IgH genes (Figure 1(F)) and IGKV1-IGKJ2 for IgK genes (Figure 1(G)). These results demonstrated that Omicron BA.1 breakthrough infection activated multiple memory B cell clonotypes for generating multiple bNAbs.

### Multiple bNAbs display distinct neutralization profiles against SARS-CoV-2 variants

**Figure 2.**
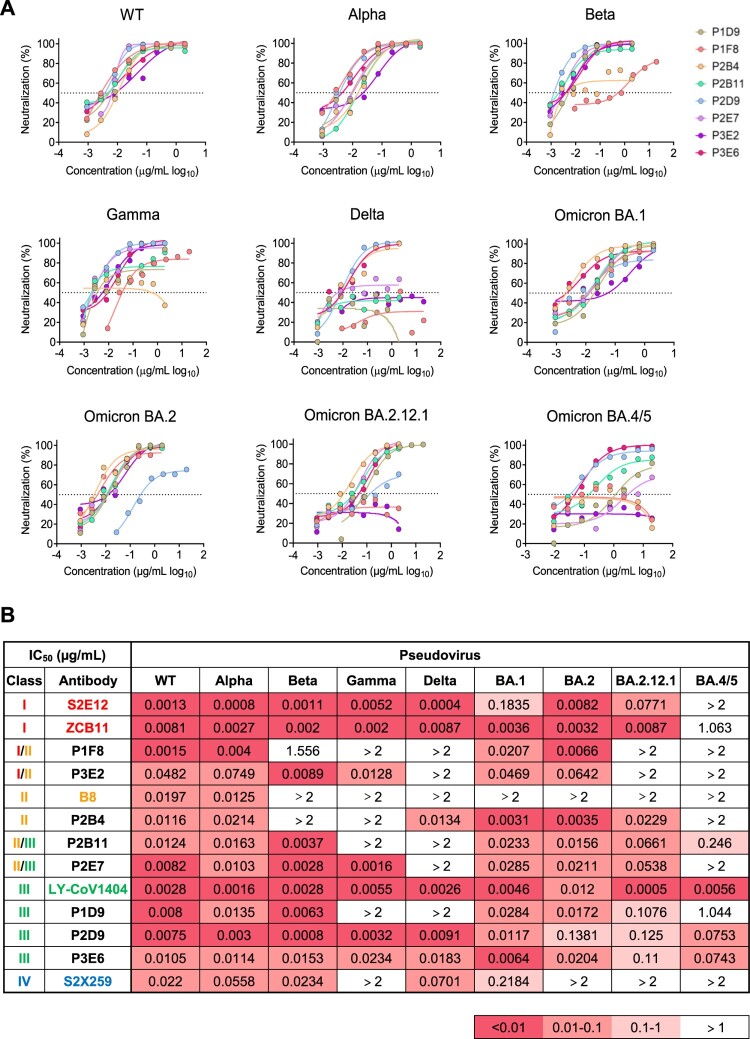
Multiple bNAbs display distinct neutralization profiles against pseudotyped SARS-CoV-2 variants. (A) The neutralization activity of P1D9, P1F8, P2B4, P2B11, P2D9, P2E7, P3E2 and P3E6 against pseudotyped SARS-CoV-2 WT and variants. The dashed line in each graph indicates 50% neutralization. (B) The summary of IC_50_ of the above NAbs. Published NAb controls from class I-IV (S2E12, ZCB11, B8, LY-CoV1404 and S2X259) are highlighted in colour. Ranges are indicated according to the colour bar below.

**Figure 3.**
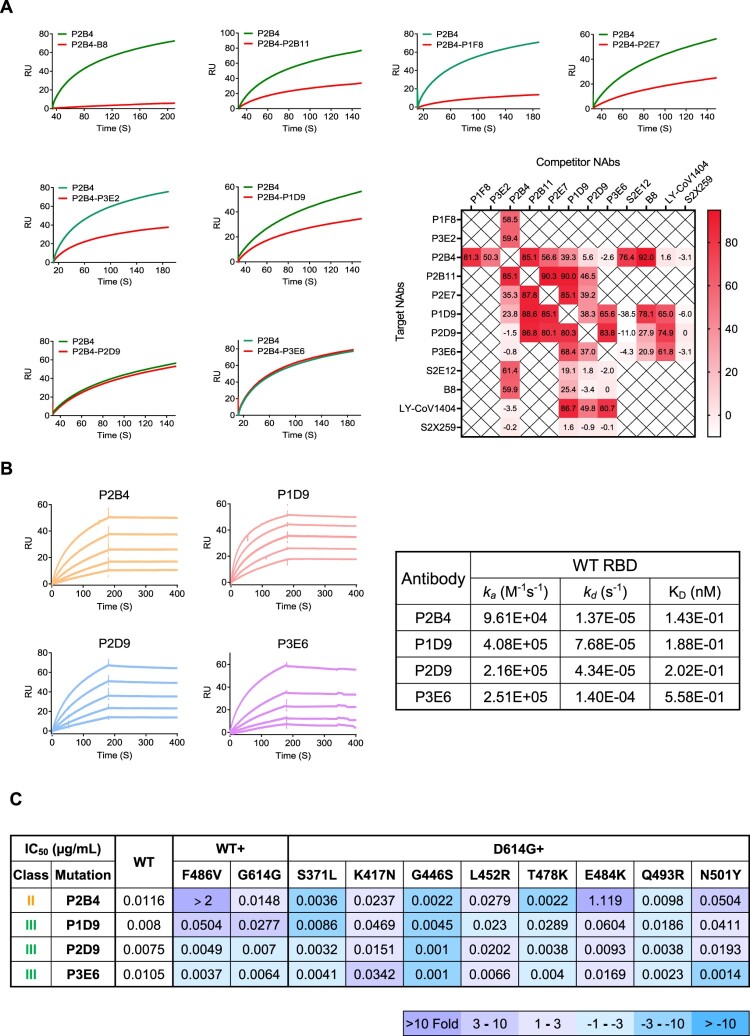
Surface plasmon resonance analysis reveals binding epitopes of newly identified bNAbs. (A) Competitive binding between NAbs to SARS-CoV-2 WT RBD. The curves show the binding of P2B4 from class II to RBD without (green) or with (red) pro-incubation of competitor NAbs including B8 as control. The heatmap summarizes the ratio of response units (RU) difference between indicated antibodies including controls, expressed as a percentage. The time point of RU for analysis is when the binding steady state was reached. No competitive binding (<30%), partially competitive binding (30-80%) and totally competitive binding (>80%) are indicated with gradient colour. (B) Binding kinetics of P2B4, P1D9, P2D9 and P3E6 to SARS-CoV-2 WT RBD was determined by SPR. (C) The summary IC_50_ of P2B4, P1D9, P2D9 and P3E6 against pseudotyped SARS-CoV-2 WT with F486V, D614G or D614G plus the indicated point substitutions found within the RBD. Fold change of IC50 values relative to WT or D614G are indicated according to the colour bar below.

To evaluate the potency and breadth of 21 newly identified NAbs with strong neutralization activity, we tested them against a panel of SARS-CoV-2 pseudoviral variants including VOCs and multiple Omicron sub-lineages (Figure 2, Figure S4 and Table S2). Previously reported class I S2E12 as well as ZCB11, class II B8, class III LY-COV1404 (Bebtelovimab) and class IV S2X259 were included as NAb controls (Figure S5) [21–23]. We found that 8 (8/21, 38%) NAbs (P1D9, P1F8, P2B4, P2B11, P2D9, P2E7, P3E2, P3E6) displayed potent neutralization against not only WT at a mean IC_50_ value of 13.5 ng/mL (range 1.5-48.2 ng/mL) but also BA.1 at a similar mean IC_50_ value of 21.1 ng/mL (range 3.1-46.9 ng/mL) (Figure 2(B)). For other VOCs and Omicron sub-lineages tested, however, each of these NAb exhibited a quite distinct neutralization profile. For example, significantly reduced neutralization was found for P1D9 against Gamma, Delta and Omicron BA.4/5, and for P2B4 against Beta, Gamma and Omicron BA.4/5. Notably, P2D9 and P3E6 neutralized all variants tested with slightly reduced activities against Omicron BA.2.12.1 and BA.4/5 at a mean IC_50_ value of 41.5 (range 0.8–138.1 ng/mL) and 34.9 ng/mL (range 6.4–110 ng/mL), respectively. These results demonstrated that each of these broadly NAbs (bNAbs) displayed distinct potency and breadth against the panel of viruses tested. Besides the sequence diversity in these antibody genes, immune escape mutations found in the viral spike were associated with the reduced neutralization potency and breadth as indicated in recent publications [7,9,11]. Based on our results, the mutations in BA.2.12.1 (e.g. L452Q) might account for resistance to P1F8 and P3E2. The mutations in BA.4/5 (e.g. F486V) might account for resistance to P2B4 and P2E7. P2D9 and P3E6, however, remained potent against BA.4/BA.5 with IC_50_ values at 70 ng/mL. Although the mutation F486R in BA.4/5 resulted in resistance to class I bNAb ZCB11, it did not seem to affect P2D9 and P3E6 (Figure 3(C)). These results demonstrated that newly identified bNAbs might engage distinct modes of action underlying their diverse neutralization breadth and potency.

### Surface plasmon resonance analysis reveals binding epitopes of newly identified bNAbs

To determine the binding properties of these 8 potent bNAbs, we performed a competition-binding assay for their interaction with viral RBD using the surface plasmon resonance (SPR) analysis (Figure 3(A), Figures S6 and S7). Control antibodies included class I S2E12, class II B8, class III LY-CoV1404 and class IV S2X259. The competitive binding between tested antibodies was quantified by the reduction of response units (RU) to immobilized RBD in the absence and presence of the potential competitor. We found that P2B4 had the strongest competition with the class II B8 followed by class I S2E12. P2B4 then competed strongly with P1F8 and P2B11 followed by P2E7 and P3E2, but hardly with class III LY-CoV1404 and class IV S2X259, suggesting a class II phenotype. In contrast, P2B4 did not compete with P1D9, P2D9 and P3E6. Instead, P1D9, P2D9 and P3E6 compete strongly with class III LY-CoV1404 but neither with class I S2E12 nor with class IV S2X259, respectively. P2B11 and P2E7 compete strongly with P1D9 and P2D9 as well. By analysing the complete set of profiles of our eight bNAbs in competition with all control antibodies tested, we estimated that P1F8, P3E2 and P2B4 are likely class I/II NAbs, whereas P1D9, P2D9 and P3E6 belong to class III NAbs. P2B11 and P2E7 are intermediate class II/III NAbs due to strong competition with P1D9 and P2D9 as well.

Subsequently, class II P2B4 and class III P1D9, P2D9 and P3E6 bNAbs were subjected to a more detailed analysis due to their potency and breadth against Omicron variants (Figure 2(B)). We found that all of them showed high-affinity binding to WT RBD at sub-nM concentrations (Figure 3(B)). As depicted above (Figure 2(B)), P2B4 exhibited the highest potency against Omicron BA.1 and BA.2 with IC_50_ values of 3.1 and 3.5 ng/mL, respectively. P2B4, however, lost its neutralization activity substantially against Beta, Gamma and Omicron BA.4/5. By testing a panel of pseudoviruses containing individual mutations of antibody evasion (Figure 3(C) and Figure S8), we found that F486V in BA.4/5 and E484K in Beta and Gamma were the key neutralization resistant mutations for P2B4. The L452R mutation in Delta and BA.4/5 showed less than 3-fold neutralization resistance to P2B4, explaining its retained potency against Delta. For class III bNAbs P1D9, P2D9 and P3E6, individual mutations including L452R and F486V in BA.4/5, did not seem to result in substantial neutralization resistance. These results indicated that class III P1D9, P2D9 and P3E6 might have higher resistant barrier than class II P2B4, which required further structural analysis.

### Structural basis of class II NAb P2B4 for viral neutralization and escape

**Figure 4.**
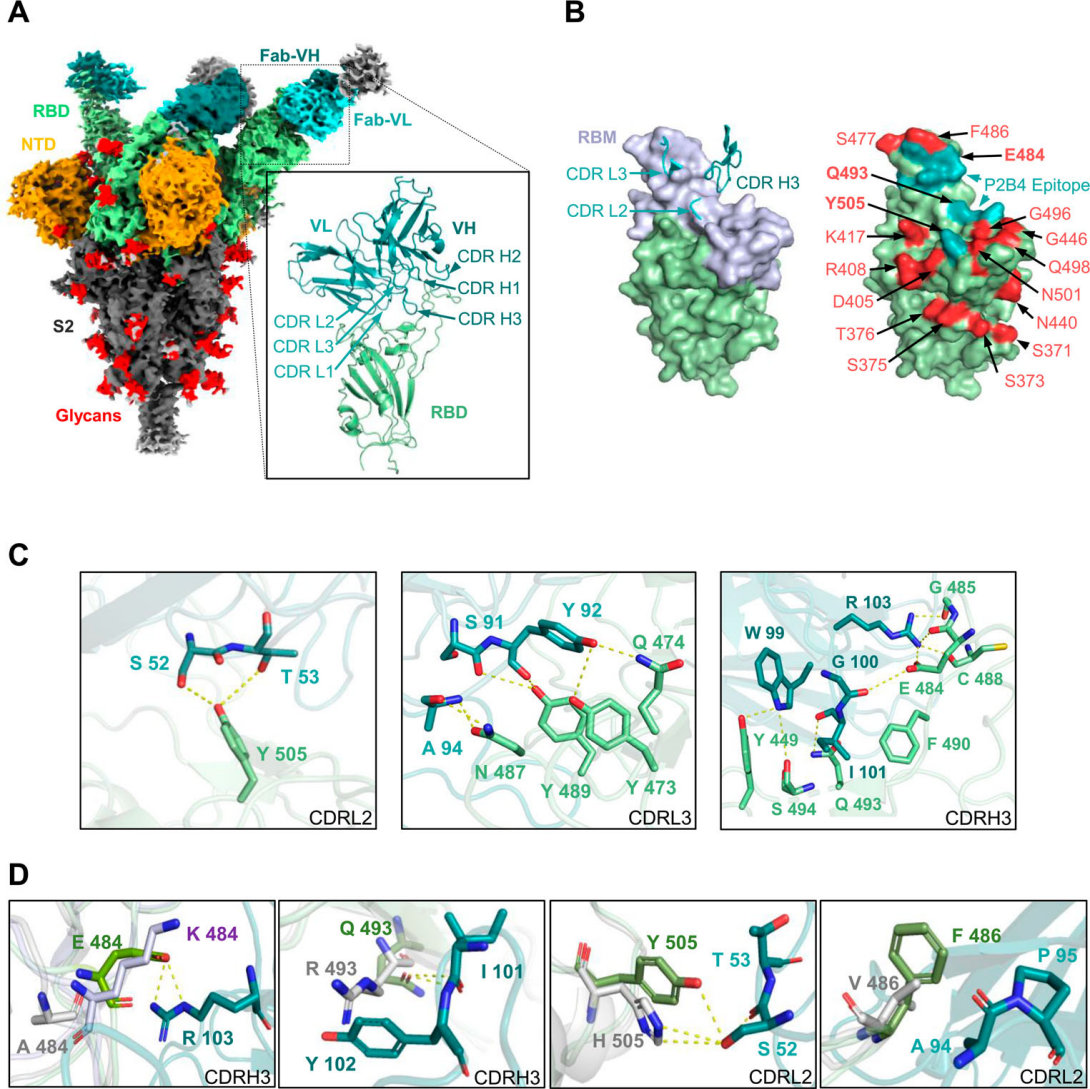
Structural basis of class II NAb P2B4 for viral neutralization and escape. (A) Cryo-EM density map of SARS-CoV-2 WT spike in complex with P2B4 Fab. NTD is shown in orange, RBD in green, glycans in red, with Fab heavy chain in dark cyan and light chain in light cyan. The cartoon represents the structure of P2B4 variable regions binding WT RBD after local focused refinement. The variable region of light chain (VL), of heavy chain (VH) and RBD are coloured light cyan, dark cyan and olive, respectively. (B) P2B4 CDR L2/L3/H3 are involved in binding WT RBD (left). The receptor-binding motif (RBM) is coloured light purple. The epitope is shown in cyan on RBD surface with VOCs mutation sites coloured red (right). E484, Q493 and Y505 in bold font are included in the epitope of P2B4. (C) Structural basis of P2B4 neutralization. Residues of CDRs and RBD involved in interaction are labelled in light cyan (CDR L2/L3), dark cyan (CDR H3) and olive (RBD), respectively. Hydrogen bonds and salt bridges are represented by yellow dashed lines. (D) Structural basis of P2B4 evasion. Mutations in E484, Q493 and Y505 are labelled in P2B4 Fab-WT RBD complex (P2B4 in cyan and RBD in green) aligned with Beta RBD (PDB:7VX4, light purple) or Omicron BA.1 RBD (PDB:7TLY, grey). F486 in BA.1 RBD is substituted into valine to structurally simulate BA.4/5. Hydrogen bonds are represented by yellow dashed lines.

To understand the molecular basis of P2B4 neutralization and antibody evasion, the cryo-EM structure of WT 2P spike in complex with P2B4 Fab was determined at 3.8 Å resolution (Figure S9 and Table S3). We further used local refinement with particle subtraction to obtain a high-quality reconstruction for the P2B4-RBD interface at 3.5 Å resolution (Figure 4(A), inset). Encoded by IGKV1-39 and IGHV3-9 genes (Table S6), two P2B4 Fabs bound to up RBDs with the third Fab binding to a down RBD on the spike trimer (Figure 4(A)), indicating the 2-up 1-down (2u1d) feature of class II NAbs [24]. Consistent with membership in class II B8, [25], the light chain interface of P2B4 involved CDR L2 and CDR L3 but not CDR L1 (Figure 4(B), left). In addition, the 17-residue long CDR H3 of P2B4 inserted into the central ridge area of the receptor-binding motif (RBM) in RBD that would block cellular receptor ACE2 binding [26]. Some P2B4 binding residues S52 and T53 in CDR L2, as well as G100, I101 and R103 in CDR H3 were in the interface potentially interacting with VOC-associated residues E484, Q493 and Y505 (Figure 4(B), right, (C)). For example, Q493R in BA.1 and BA.2 significantly reduced class II LY-CoV555 (Bamlanivimab) neutralization against Omicron BA.1 [27]. This mutation, however, did not affect P2B4 neutralization potency (Figure 3(C)) and R493 did not form the hydrogen bond to I101 in CDR H3 (Figure 4(D)). In contrast, the Fab structures aligned with VOC RBDs [28,29] revealed that E484A in Omicron variants or E484K in Beta/Gamma eliminated the salt bridge interaction with R103 in CDR H3 (Figure 4(D)), resulting in significant loss of neutralization against Beta/Gamma (Figure 2(B) and Figure 3(C)). Y505H in all Omicron sub-lineages might create additional hydrogen bonds with S52 in CDR L2, compensating for some lost interaction with E484A and Q493R, explaining the conservation of P2B4 neutralization against BA.1 and BA.2. Similar to class I ZCB11 [21] and class II LY-CoV555 [27], F486V was the key mutation in BA.4/5 near the CDR L3-RBD interface (Figure 4(D)), accounting for complete loss of neutralization (Figure 3(C)). Since V486 with smaller side chain did not seem to impact antibody interaction directly, this mutation might alter overall RBD structural conformation, destroying the hydrophobic environment at the spike-antibody interface.

### Structural basis of class III P1D9 and P2D9 for viral neutralization and escape

**Figure 5.**
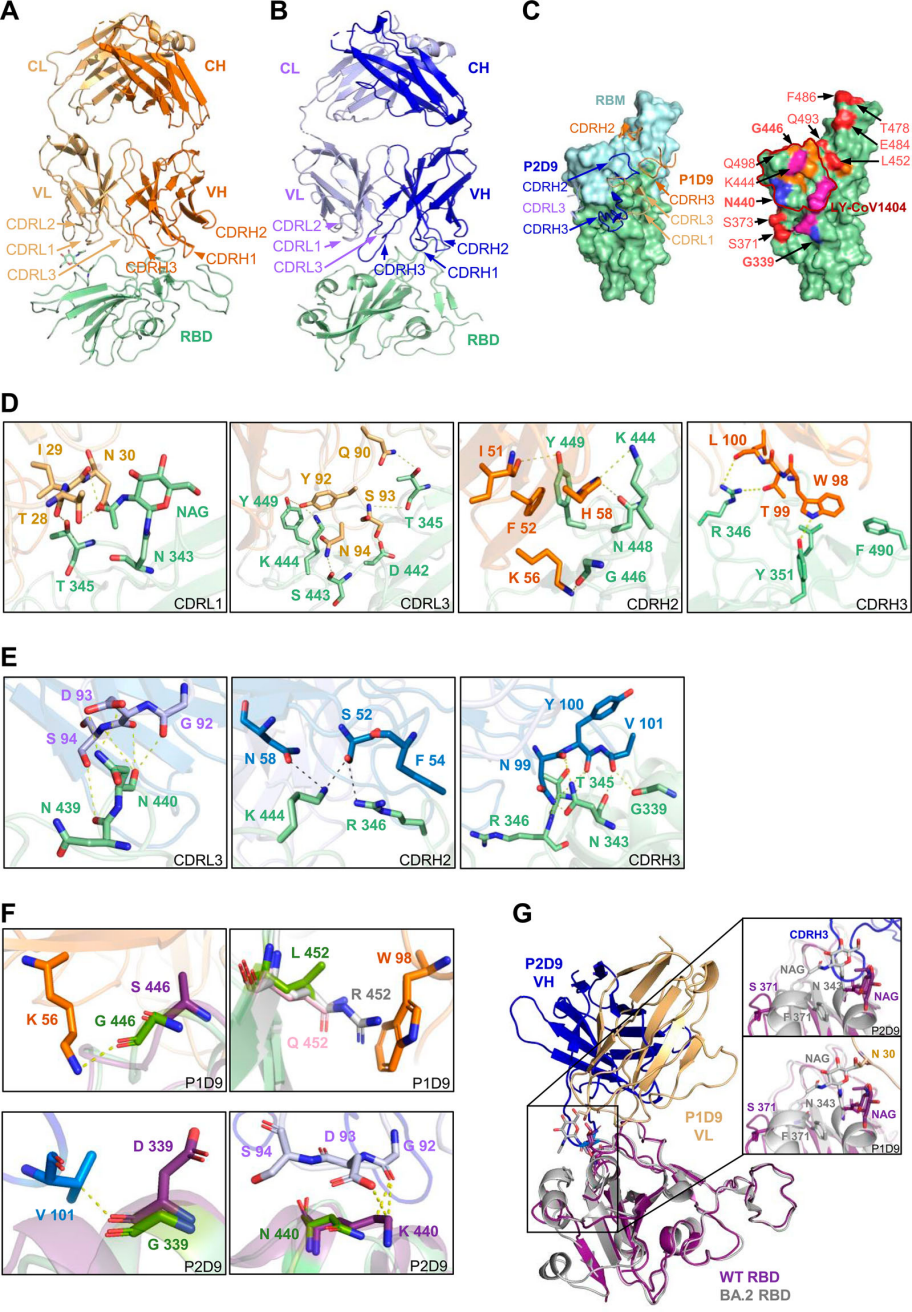
Structural basis of class III P1D9 and P2D9 for viral neutralization and escape. (A) The cartoon represents the structure of P1D9 variable regions binding SARS-CoV-2 WT RBD after local focused refinement. The variable region of light chain (VL), of heavy chain (VH) and RBD are coloured light yellow, orange and olive, respectively. (B) The cartoon represents the structure of P2D9 variable regions binding SARS-CoV-2 WT RBD after local focused refinement. VL, VH and RBD are coloured violet, blue and olive, respectively. (C) P1D9 CDR L1/L3/ H2/H3 and P2D9 CDR L3/H2/H3 are involved in binding WT RBD (left). RBM is coloured light blue. P1D9, P2D9 and their overlapped epitope are shown in orange, blue and magenta on the RBD surface, respectively (right). VOCs mutation sites are coloured red, and G446, G339 as well as N440 included in the epitope are in bold font. The epitope of published NAb LY-CoV1404 is labelled in brown. (D) Structural basis of P1D9 neutralization. Residues of CDR and RBD involved in interaction are labelled in light yellow (CDR L1/L3), orange (CDR H2/H3) and olive, respectively. Hydrogen bonds and salt bridges are represented by yellow dashed lines. (E) Structural basis of P2D9 neutralization. Residues of CDR and RBD involved in interaction are labelled in violet (CDR L3), blue (CDR H2/H3) and olive, respectively. Hydrogen bonds and salt bridges are represented by dashed lines. (F) Structural basis of P1D9 and P2D9 evasion. Mutations in G446 and L452 are labelled in P1D9 Fab-WT RBD complex (P1D9 in orange and RBD in green) aligned with Omicron BA.1 RBD (PDB:7TLY, purple) or Delta RBD (PDB:7W9F, grey) (Top). L452 in WT RBD is also substituted into Glutamine (pink) to structurally simulate BA.2.12.1. Mutations in G339 and N440 are labelled in P2D9 Fab-WT RBD complex (P2D9 VH in blue, VL in violet and WT RBD in green) aligned with BA.1 RBD (PDB:7TLY, purple) (bottom). Hydrogen bonds are represented by yellow dashed lines. (G) Structural overlay of P1D9/P2D9 Fab-WT RBD (purple) complexes with BA.2 RBD (PDB:7UB6, grey). The 366–377 hairpin and N343 glycan in RBD interacting with P1D9 CDR L1 coloured light yellow and P2D9 CDR H3 coloured blue are zoomed in.

To understand the different neutralization profiles of class III P1D9 and P2D9, we obtained the crystal structures of WT RBD-P1D9 Fab and WT RBD-P2D9 Fab complexes with resolutions of 2.8 and 3.9 Å, respectively (Figure 5(A, B) and Table S4). Both P1D9 and P2D9 used the same IGHV1-69*06 but different IGKV1-39*01 and IGKV3-20*01 (Table S6), respectively, involving both heavy chain and light chain to target the RBD at different angles (Figure 5(A, B)). The structures revealed that both antibodies interacted with RBM residues mostly outside the ACE2-binding site (Figure 5(C), left). RBD binding of P1D9 involved CDR L1, CDR L3, CDR H2 and CDR H3, with its footprint shifted upward on the RBD backside compared to P2D9, which used CDR L3, CDR H2 and CDR H3 (Figure 5(C), left). The binding residues of P1D9 included T28, I29 and N30 in CDR L1, Q90, Y92, S93 and N94 in CDR L3, I51, K56 and H58 in CDR H2, as well as W98, T99 and L100 in CDR H3, interacting with RBD residues including T345, N343, T345, R346, Y351, D442, S443, K444, G446, N448 and Y449 (Figure 5(D)). The binding residues of P2D9 included G92, D93, S94 in CDR L3, S52 and N58 in CDR H2, and N99, Y100 and V101 in CDR H3, interacting with RBD residues including G339, N343, T345, R346, N439, N440 and K444 (Figure 5(E)). Clearly, the RBD binding epitope of P1D9 overlapped with that of P2D9, but they were not identical (Figure 5(C), right). We then tested possible individual RBD residues that might confer P1D9 resistance against Gamma, Delta and BA.4/5 (Figure 3(C)). G446S in BA.1 significantly reduced the neutralization of NAb A19-61.1 by forming a steric clash [27]. This mutation, however, did not introduce a major clash for P1D9 although it destroyed the hydrogen bond with K56 in CDR H2 (Figure 5(F), top left). L452R in Delta [30] and BA.4/5 caused evasion to many class III NAbs according to recent reports [7,9,31]. In the aligned structure, Q452 in BA.2.12.1 and R452 in BA.4/5 potentially formed steric hindrance or a clash with W98 in CDR H3, respectively (Figure 5(F), top right), although this did not significantly impact the potency of P1D9 (Figure 3(C)). It is conceivable that combined mutations such as K417N, E484K and N501Y in Gamma, as well as F486V and N501Y in BA.4/5 accounted jointly for the major neutralization resistance of P1D9 to these VOCs compared with WT (Figure 3(C)). In contrast, the potency of P2D9 was not affected by F486V alone, similar to some class III antibodies (e.g. LY-CoV1404 and S309) [9], and therefore could more potently neutralize BA.4/5 compared with P1D9. Aligned structures indicated that viral G339D and N440K mutations could form new hydrogen bonds with V101 in CDR H3 and G92 in CDR L3 of P2D9 (Figure 5(F), bottom), contributing to its neutralization potency against Omicron variants. Notably, S371L, S373P and S375F mutations in BA.1 reduced the binding between NAb S309 and the viral glycan on N343 [27]. Similarly, our P1D9 Fab- and P2D9 Fab-WT RBD complex aligned with BA.2 RBD [32] revealed that the loop conformation could be changed by two new mutations S371F and T376A found in BA.2, BA.2.12.1 and BA.4/5, resulting in N343 glycan movement (Figure 5(G)). The fucose moiety of this glycan, in turn, clashed with the 17-residue long CDR H3 of P2D9 directly while maintaining a short distance from N30 of P1D9 (Figure 5(G), insets). The heavy chain of P2D9 might be pushed upward in the context of mutant RBDs, which might explain its relatively reduced neutralization activities against BA.2, BA.2.12.1 and BA.4/5 (Figure 2(B)).

### Unique interaction between class III P3E6 Fab and Omicron BA.1 spike

**Figure 6.**
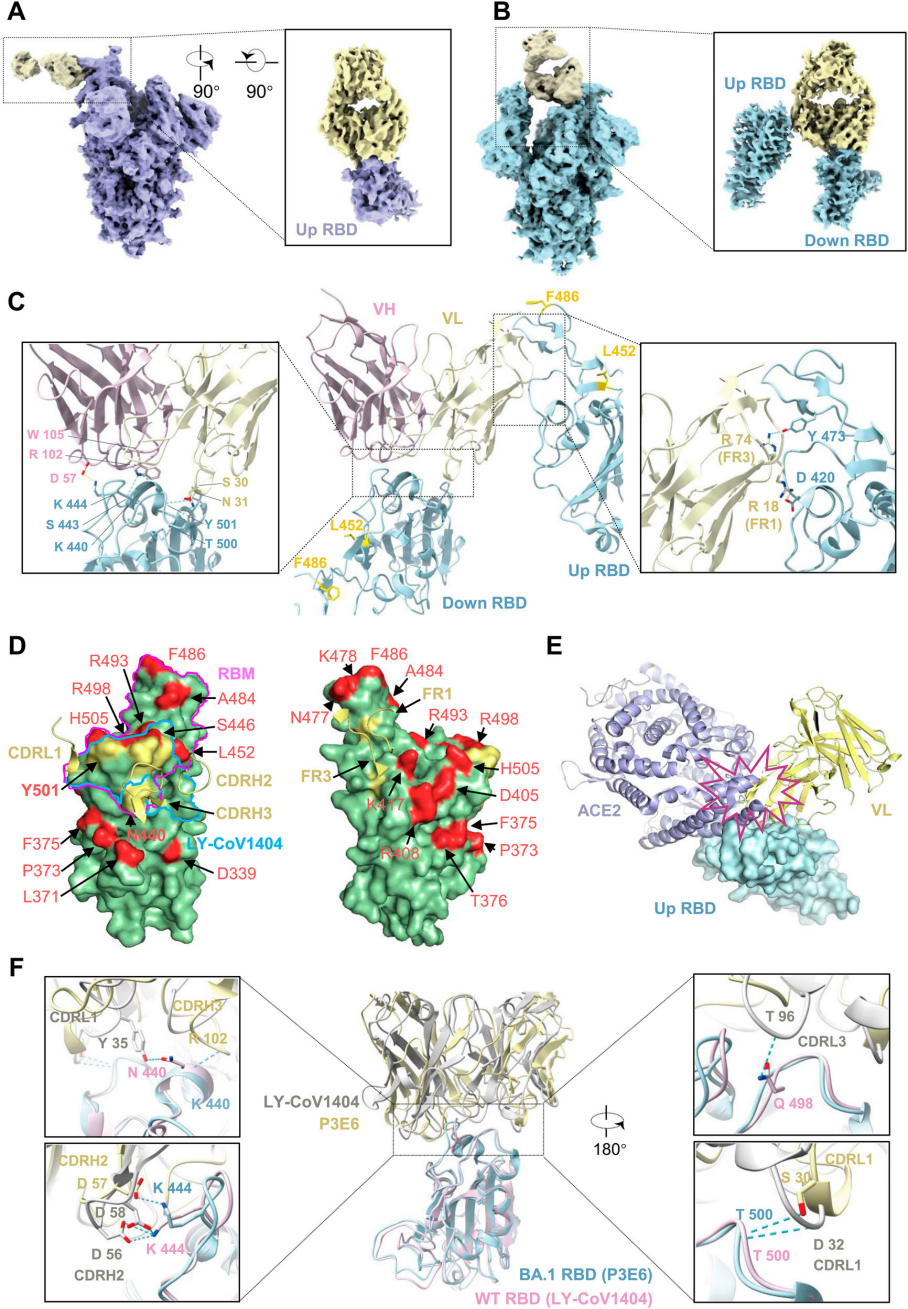
Unique interaction between class III P3E6 Fab and Omicron BA.1 spike. (A and B) Cryo-EM density map of SARS-CoV-2 Omicron BA.1 spike in complex with P3E6 Fab. Two different binding modes of P3E6 are shown. Spike trimer with one up and two down RBDs (1u2d) is coloured purple (A), as well as with two up and one down RBDs (2u1d) is coloured blue (B), respectively. P3E6 Fab is coloured yellow. (C) Interaction between P3E6 Fab and BA.1 RBD. P3E6 Fab is shown as the variable region of heavy chain (VH, pink) and light chain (VL, light yellow), and RBDs in up or down conformation are coloured blue. Two interfaces are zoomed in to show key residues contributing to the interaction. (D) P3E6 CDR L1/H2/H3 are involved in binding BA.1 RBD (left). The epitope is shown in light yellow on the RBD surface. VOCs mutation sites are coloured red, and K440 as well as Y501 included in the epitope are in bold font. RBM and the epitope of published NAb LY-CoV1404 are labelled in magenta and blue, respectively. FR1/FR2 of P3E6 light chain interact with a separate up RBD (related to c right) and contribute to stabilizing the structure (right). (E) Binding of P3E6 light chain to an up RBD (related to C right) prevents binding of the ACE2 receptor. ACE2 and the VL are coloured purple and light yellow, respectively. (F) Comparison between the binding pattern of P3E6 (light yellow) in complex with BA.1 RBD (light blue) and LY-CoV1404 (PDB: 7MMO, grey) in complex with WT RBD (pink). The residues involved in the interaction are labelled.

To determine the mode of P3E6 action at the molecular level, we also solved the complex structure of P3E6 Fab bound to Omicron BA.1 spike trimer by singe-particle cryo-EM at 3.2 Å resolution (Figure S10A, B and Table S5). 3D classification identified two major types of the spike-P3E6 complex, each with a similar number of particles (Figure S10C). The first type showed that P3E6 bound only to the up RBD of the spike trimer with a conformation of 1 up and 2 down RBDs (1u2d) (Figure 6(A)). The second type, however, adopted a 2u1d conformation (Figure 6(B)). Unlike some previously characterized class III NAbs, while the down RBD contributed to binding primarily, there was a second interaction between P3E6 and one up RBD, establishing a unique bivalent binding mode. These results demonstrated that P3E6 is a class III bNAb using distinct bivalent interactions with both up and down RBDs [24]. Furthermore, to reveal residues at the binding interfaces, a mask covering P3E6 and its interacting RBD was applied to the two aforementioned types to improve local resolution (Figure S10C). We found that the binding epitopes of P3E6 in the up RBD from type I and in the down RBD from type II were nearly identical. The mutual epitope contained residues from both heavy and light chains of P3E6. The interaction of P3E6 with RBD was stabilized mainly by three forces including (1) hydrogen bonds formed between K440, S443, T500, Y501 of RBD and R102, W105 in CDR H3 plus S30, N31 in CDR L1, (2) salt bridge between K444 of RBD and D57 in CDR H2, and (3) hydrophobic interaction between V445 of RBD and W105 in CDR H3 (Figure 6(C), left). Since the mutual epitope of P3E6 was partially overlapped with the binding surface of ACE2 (Figure 6(D), left), P3E6 acted like LY-CoV1404 as a competitive antibody by blocking binding of ACE2 to the viral spike [22]. Intriguingly, when P3E6 bound to the down RBD (type II), the interaction was also stabilized by another interface with a separate up RBD. In this interface, two arginine residues (R18 of FR1 and R74 of FR3) in the P3E6 light chain formed a salt bridge with D420 and a hydrogen bond with Y473 of RBD, respectively (Figure 6(C), right, (D), right). Besides, docking RBD-bound ACE2 to the P3E6-up RBD complex indicated a steric clash, and therefore contributed to blocking of ACE2 binding (Figure 6(E)). Since the map quality of this interface was relatively lower than the mutual interface, it likely represents a flexible/dynamic binding mode. Nevertheless, the bivalent binding mode of P3E6 is unique and has not been described previously. Notably, the epitope of P3E6 was mainly located on a mutation-free RDB surface with edges contacting a few substitutions (K440 and Y501; Figure 6(D), left). Structural analysis showed that L452R and F486V, two unique mutations in BA.4/5 variants, were distant from these interacting interfaces (Figure 6(C)), explaining the high potency of P3E6 compared with P1D9. Further structural comparison of spike trimer bound with P3E6 or LY-CoV1404 revealed divergences in their binding modes. Compared to P3E6, LY-CoV1404 presented apparently tighter binding to RBD with more residues involved to form a relatively stronger interaction network including hydrogen bonds and salt bridges [22] (Figure 6(F)). This could be one of the reasons why LY-CoV1404 displayed higher potency than P3E6.

## Discussion

The continuously emerged SARS-CoV-2 variants have overwhelmingly outcompeted the speed of new COVID-19 vaccine and antibody development. From the end of 2021, several new waves of global infections have resulted from Omicron sub-lineages including BA.1, BA.2, BA.2.12.1 and BA.4/5. Each sub-lineage has evidently become more resistant to vaccine-induced neutralizing antibodies, even leading to antigenic shift [7-9]. Understanding hybrid immunity generated among vaccinees with breakthrough infections becomes essential for determining the magnitude, breadth and potency of neutralizing antibody responses against SARS-CoV-2 Omicron variants. In this study, we investigated the types of memory B cell responses generated during hybrid immunity in mRNA-vaccinee after breakthrough infection by SARS-CoV-2 Omicron BA.1. [14]. By analysing 104 single memory B cell-derived mAbs, we found that the BA.1 infection effectively recalled diverse B cell clonotypes and Ig genes activated to generate mainly cross-reactive bNAbs (60%) besides WT- and BA.1-specific NAbs. Majority of these neutralizing antibodies (24/35, 68.6%) were RBD-specific. Notably, the most potent eight NAbs consisted of both class I/II and class III potent bNAbs. Although these bNAbs involved diverse IgH and IgL sequences and binding epitopes, they exhibited high and comparable potency against both WT and BA.1 likely due to specific and mutual antigens in the mRNA vaccine and the homogenous breakthrough BA.1 strain. We then demonstrated that these bNAbs displayed diverse neutralizing activities against other VOCs and Omicron sub-lineages due to their sequence and structural differences. Two class III bNAb P2D9 and P3E6 were able to neutralize all VOCs and Omicron sub-lineages tested including the rising BA.4/5 strains. Structural analysis consistently revealed diverse modes of bNAb action due to their distinct binding footprints on viral RBD or spike proteins. Our findings, therefore, add new insights into hybrid immunity generated by mRNA vaccine-induced diverse memory B cells in response to Omicron breakthrough infection, which may have implications for implementing public measures of population protection and next-generation vaccine development.

BA.1 breakthrough infection recalled broad memory B cell responses induced by the mRNA vaccine for generating diverse bNAbs. We and others have previously reported that Omicron breakthrough infection after 2 doses of mRNA vaccine appeared to recall cross-reactive NAbs within ten days against current VOCs, including Alpha, Beta, Gamma, Delta and Omicron, from unmeasurable IC_50_ values to high NAb titres, which were better than the mean peak IC_50_ values of BioNTech-vaccinees [7,8,14]. The quickly recalled bNAb responses, together with spike- and NP-specific T cells might contribute to disease progression control [14,33]. However, the clonotype and diversity of memory B cells as well as the breadth of bNAbs elicited during the infection-boosted responses remains incompletely understood. We found that the BA.1 breakthrough infection in fact was able to activate divergent IgG genes to generate a pool of diverse antibody clonotypes (Figure 1(B, D–G) and Table S6). IGHV1-69 is likely the predominant B cell clonotype among isolated NAbs after the BA.1 breakthrough infection like recent publications [18,19], whereas other IGHV genes 3–53, 3–49 and 3–23 were also involved. Since the infection was caused by a homogenous BA.1 strain as previously reported [15], it is conceivable that the BA.1-based booster vaccines under clinical trials might have the potential to generate similar bNAbs among mRNA vaccinees. Inducing bNAbs is critical because we consistently found here that immune escape mutations found in the viral spike were associated with reduced neutralization potency and breadth, which would affect vaccine efficacy as well [7,9,11]. Besides BA.1-based vaccines, it is also conceivable that hybrid immunity-generated multiple potent bNAbs with non-overlapping binding epitopes of both class I/II and III characteristics would be beneficial for broad protection against Omicron variants.

The structural analysis confirmed that bNAbs in the same class could target at multiple distinct epitopes. Unlike the class II NAb B8 which has quite limited breadth, the class II bNAb P2B4 could neutralize most VOCs potently, except for Beta, Gamma and BA.4/5, by blocking the receptor-binding domain (RBM). The 3D structure of Fab-RBD interface and pseudovirus assay indicated that E484K and N501Y, the mutations in Beta and Gamma, led to 75-fold and 3.4-fold increase in P2B4 resistance, respectively. Those mutations conferred high resistance to several RBD-specific class I/II NAbs such as B8, CB6, LYCoV555 and 2–15 [11,27,34]. Instead, F486V found in BA.4/5, which could increase the binding affinity of BA.4/5 to ACE2 [35], likely accounted for P2B4 resistance although the mechanism remains to be investigated. Then, each of the class III P1D9, P2D9 and P3E6 relied on different residues in CDR H2/H3/L1/L3 to target distinct epitopes as their variable region shared about 50% sequence identity (Figure S11). Their epitopes were partially overlapping, resulting in the observed bNAb competition, but not identical to one another or to those previously described for other class III bNAb (e.g. LY-CoV1404). Although their epitopes were located mainly on a mutation-free surface, neutralization was clearly interfered by some substitutions. The L452R mutation in Delta and BA.4/5 or the L452Q mutation in BA.2.12.1 contributed to significant P1D9 resistance by forming a potential steric clash. S371F and T376A mutations in BA.2 and BA.4/5 sub-lineages could shift the position of N343 glycan nearby (Figure 5(G)), and therefore, conferred partial P2D9 resistance. Interestingly, P3E6 neutralizes all variants tested by engaging unique bivalent interactions with two RBDs, which has not been reported previously. Since P2D9 and P3E6 displayed similar potency against recently emerged BA.4/BA.5, the breadth of the hybrid immunity would be broadly protective. To this end, the natural booster by low pathogenic Omicron variant infection would be beneficial for inducing diverse bNAbs necessary to build stronger community immunity, which may have implications for implementing public measures of population protection.

The newly emerged Omicron subvariants have escaped from many potent NAbs under clinical development, which has posed problems for industries for generating bNAb-based therapy. BA.4/5 with L452R and F486V mutations conferred substantially stronger (more than 10-fold) escape compared to BA.2, in line with viral resistance to neutralization by many clinically authorized NAbs [7,9]. Revealing the structure of four newly identified bNAbs has undoubtedly added new values for bNAb engineering for optimized potency and breadth. Conceivably, with more bNAbs structural data made available, it would enhance the power of deep learning-guided optimization of human bNAbs by targeting highly conserved epitopes [36], which have implications not only to overcome naturally emerged escape viral mutations but also to generate ultrapotent and broadly reactive bi- or tri-specific bNAb for clinical immunotherapy.

## Materials and methods

### Ethics

This acquisition of blood samples from a vaccinated patient for identification of broad neutralizing activities and isolation of potent monoclonal antibodies against COVID-19 received approval from the Institutional Review Board of The University of Hong Kong/Hospital Authority Hong Kong West Cluster (Ref No. UW 21-452). The research was conducted in strict accordance with the rules and regulations of the Hong Kong government for the protection of human subjects. The study subjects agreed and signed the written informed consents for research use of their blood samples and indirect identifiers.

### Human subject

An Omicron BA.1 case-patient who received two doses of BNT162b2 around 180 days before symptom onset was recruited for voluntary blood donation [14]. Clinical and laboratory findings were entered into a predesigned database. Written informed consent was obtained from all study subjects. Blood samples were collected by professional clinical doctors and separated into plasma and peripheral blood mononuclear cells (PBMCs) by Ficoll-Hypaque gradient centrifugation. All plasma samples were heat-inactivated at 56°C for at least 30 min before test.

### Live viruses

Authentic SARS-CoV-2 included D614G (MT835143), Delta (hCoV-19/Hong Kong/HKU-210804-001/2021; GISAID: EPI_ISL_3221329), Omicron BA.1 (hCoV-19/HongKong/HKU691/2021 (HKU691); GISAID: EPI_ISL_7138045), Omicron BA.2 (GISAID: EPI_ISL_9845731), Omicron BA.2.12.1 (hCoV-19/Hong Kong/HKU-220712-006/2022; GISAID: EPI_ISL_13777659), Omicron BA.4.1 (hCoV-19/Hong Kong/HKU-220712-004/2022; GISAID: EPI_ISL_13777657) and Omicron BA.5.2 (hCoV-19/Hong Kong/HKU-220712-005/2022; GISAID: EPI_ISL_13777658) were isolated from the combined nasopharyngeal-throat swabs of patients with COVID-19 in Hong Kong, respectively [37]. All experiments involving authentic SARS-CoV-2 followed the approved standard operating procedures in the Biosafety Level 3 (BSL-3) [38,39].

### Cell lines

Cell lines were maintained as previously described [21]. HEK293T cells, HEK293T-hACE2 cells, and Vero-E6-TMPRSS2 cells were cultured in DMEM containing 10% FBS, 2 mM L-glutamine, 100 U/mL penicillin and incubated at 37°C in a 5% CO_2_ setting [40]. Expi293FTM cells were cultured in Expi293TM Expression Medium (Thermo Fisher Scientific) at 37°C in an incubator with 80% relative humidity and a 5% CO2 setting on an orbital shaker platform at 125 ± 5 rpm/min (New Brunswick Innova™ 2100) according to the manufacturer’s instructions.

### ELISA analysis of plasma and antibody binding to trimeric spike

ELISA was performed as previously described [21]. The recombinant trimeric spike proteins derived from SARS-CoV-2 (Sino Biological) were diluted to final concentrations of 1 μg/mL, then coated onto 96- well plates (Corning 3690) and incubated at 4°C overnight. Plates were washed with PBST (PBS containing 0.05% Tween-20) and blocked with blocking buffer (PBS containing 5% skim milk or 1% BSA) at 37°C for 1 h. Serially diluted plasma samples or isolated monoclonal antibodies were added to the plates and incubated at 37°C for 1 h. Wells were then incubated with a secondary goat anti-human IgG labelled with horseradish peroxidase (HRP) (1:5000 Invitrogen) TMB substrate (SIGMA). Optical density (OD) at 450 nm was measured by SkanIt RE6.1 with VARIOSKAN Lux (Thermo Scientific).

### Isolation of SARS-CoV-2 RBD/spike-specific IgG+ single memory B cells by FACS

RBD/spike-specific single B cells were sorted as previously described [21,41]. In brief, PBMCs from vaccinated patient were collected and incubated with an antibody cocktail and a His-tagged SARS-CoV-2 RBD/spike (Sino Biological) proteins mixture for identification of RBD/spike-specific B cells. The cocktail consisted of the Zombie viability dye (Biolegend), CD19- Percp-Cy5.5, CD3-Pacific Blue, CD14-Pacific Blue, CD56-Pacific Blue, IgM-Pacific Blue, IgD-Pacific Blue, IgG-PE, CD27-PE-Cy7 (1uL/test BD Biosciences or Biolegend) and the mixture consisted of His-tagged WT, Alpha, Beta, Gamma and Delta RBD/spike. Two consecutive staining steps were conducted: the first one used an antibody and RBD/spike cocktail incubation of 30 min at 4°C; the second staining involved staining with anti-His-APC (5 uL/test Abcam) and anti-His-FITC antibodies (1 uL/test Abcam) at 4°C for 30 min to detect the His tag of the RBD/spike. The stained cells were washed and resuspended in PBS containing 2% FBS before being strained through a 70-μm cell mesh filter (BD Biosciences). SARS-CoV-2 spike-specific single B cells were gated as CD19^+^ CD27^+^ CD3^-^CD14^-^CD56^-^IgM^-^IgD^-^IgG^+^ spike^+^ and sorted by FACSAria III cell sorter (BD) into 96-well PCR plates containing 10 μL of RNAase inhibiting RT–PCR catch buffer (1 M Tris-HCl pH 8.0, RNase inhibitor, DEPC treated water, Thermo Scientific). Plates were then snap-frozen on dry ice and stored at −80°C until the reverse transcription reaction. The population analysis of antigen-specific memory B cells was performed by FlowJo V10.

### Single B cell RT–PCR and antibody cloning

Single memory B cells isolated from PBMCs of vaccinated donors were cloned as previously described [21,42]. Briefly, one-step RT–PCR was performed on sorted single memory B cell with a gene-specific primer mix, followed by nested PCR amplifications and sequencing using the heavy chain and light chain-specific primers. Cloning PCR was then performed using heavy chain and light chain-specific primers containing specific restriction enzyme cutting sites (heavy chain, 5′-AgeI/3′-SalI; kappa chain, 5′-AgeI/3′-BsiWI). The PCR products were purified and cloned into the backbone of antibody expression vectors containing the constant regions of human Igγ1. The constructed plasmids containing paired heavy and light chain expression cassettes were co-transfected into 293 T cells (ATCC) grown in 6-well plates. Antigen-specific ELISA and pseudovirus-based neutralization assays were used to analyse the binding capacity to SARS-CoV-2 spike and the neutralization capacity of transfected culture supernatants, respectively.

### Genetic analysis of the BCR repertoire

As previously described, heavy chain and light chain germline assignment, framework region annotation, determination of somatic hypermutation (SHM) levels (in nucleotides) and CDR loop lengths (in amino acids) were performed with the aid of the NCBI IgBlast tool (https://www.ncbi.nlm.nih.gov/igblast/) [21]. Sequences were aligned using Clustal W in the BioEdit sequence analysis package (V7.2). Antibody clonotypes were defined as a set of sequences that share genetic V and J regions as well as an identical CDR3.

### Antibody production and purification

Antibodies were produced and purified as previously described [21]. The paired antibody VH/VL chains were cloned into Igγ and Igk expression vectors using T4 ligase (NEB). Antibodies produced from cell culture supernatants were purified immediately by affinity chromatography using recombinant Protein G-Agarose (Thermo Scientific) according to the manufacturer’s instructions to purify IgG. The purified antibodies were concentrated by an Amicon ultracentrifuge filter device (molecular weight cut-off 10 kDa; Millipore) to a volume of 0.2 mL in PBS (Life Technologies), and then stored at 4°C or −80°C for further characterization.

### Pseudovirus-based neutralization assay

The neutralizing activity of NAbs was determined using a pseudotype-based neutralization assay as previously described [43]. Briefly, the pseudovirus was generated by co-transfection of HEK 293 T cells with pVax-1-S-COVID19 and pNL4-3Luc_Env_Vpr, carrying the optimized spike (S) gene (QHR63250) and a human immunodeficiency virus type 1 backbone, respectively [43]. Viral supernatant was collected at 48 h post-transfection and frozen at −80°C until use. The serially diluted monoclonal antibodies or sera were incubated with 200 TCID50 of pseudovirus at 37°C for 1 h. The antibody-virus mixtures were subsequently added to pre-seeded HEK 293T-ACE2 cells. 48 h later, infected cells were lysed to measure luciferase activity using a commercial kit (Promega, Madison, WI). Half-maximal (IC_50_) or 90% (IC_90_) inhibitory concentrations of the evaluated antibody were determined by inhibitor vs. normalized response-4 Variable slope using GraphPad Prism 8 or later (GraphPad Software Inc.).

### Neutralization activity of monoclonal antibodies against authentic SARS-CoV-2

The SARS-CoV-2 focus reduction neutralization test (FRNT) was performed in a certified Biosafety level 3 laboratory as previously described [21]. The tested antibodies were serially diluted, mixed with 50 μL of SARS-CoV-2 (10^3^ focus forming unit/mL, PFU/mL) in 96-well plates, and incubated for 1 h at 37°C. Mixtures were then transferred to 96-well plates pre-seeded with 2 × 10^4^/well Vero-E6-TMPRSS2 cells and incubated at 37°C for 24 h. The culture medium was then removed and then fixed with a 4% paraformaldehyde solution for 30 min and air-dried in the BSC again. Cells were further permeabilized with 0.2% Triton X-100 and incubated with cross-reactive rabbit sera anti-SARS-CoV-2-N (1:5000) for 1 h at RT before adding an Alexa Fluor 488 goat anti-rabbit IgG (H + L) cross-adsorbed secondary antibody (1:1000 Life Technologies). The fluorescence density of SARS-CoV-2 infected cells were scanned using a Sapphire Biomolecular Imager (Azure Biosystems) and the neutralization effects were then quantified using Fiji software (NIH).

### Antibody binding kinetics and competition between antibodies measured by surface plasmon resonance (SPR)

The binding kinetics and affinity of recombinant monoclonal antibodies for the SARS-CoV-2 WT RBD protein (Sino Biological) were analysed by SPR (Biacore T200, Cytiva) as previously described [20]. Specifically, the WT RBD protein was covalently immobilized to a CM5 sensor chip via amine groups in 10 mM sodium acetate buffer (pH 5.0) for a final RU around 250. SPR assays were run at a flow rate of 10 µL/min in HEPES buffer. For conventional kinetic/dose–response, serial dilutions of monoclonal antibodies were injected across the RBD protein surface for 180 s, followed by a 900 s dissociation phase using a multi-cycle method. Remaining analytes were removed in the surface regeneration step with the injection of 10 mM glycine-HCl (pH 1.5) for 60 s at a flow rate of 30 µl/min. Kinetic analysis of each reference subtracted injection series was performed using the Biacore Insight Evaluation Software (Cytiva). All sensorgram series were fit to a 1:1 (Langmuir) binding model of interaction. Before evaluating the competition between antibodies, both the saturating binding concentrations of antibodies for the immobilized SARS-CoV-2 RBD protein were determined separately. In the competitive assay, antibodies at the saturating concentration were injected onto the chip with immobilized RBD protein for 200 s until binding steady state was reached. The other antibody also used at the saturating concentration was then injected for 200 s, followed by another 200 s of injection of antibody to ensure a saturation of the binding reaction against the immobilized RBD protein. The differences in response units between antibody injection alone and prior antibody incubation reflect the antibodies’ competitive ability by binding to the RBD protein.

### Western blot

As previously described [21], antibodies went through SDS-PAGE at the constant voltage of 120 V and then transferring at the constant current of 0.15 A. The transferred nitrocellulose membrane was removed from the transfer gel holder cassette and washed twice with distilled water for 5 min. The membrane was blocked with blocking buffer at room temperature for 30 min with continuous shaking. The membrane was subsequently washed four times with PBST for 15 min each time and then incubated with SARS-CoV-2 S1 or S2 with His tag (1:1000 Sino Biological) diluted in 5% BSA at 4°C overnight with continuous shaking. The membrane was washed four times with continuous shaking in PBST for 15 min followed by incubation with fluorescence-linked secondary antibody (1:1000 DyLight 650 rabbit anti-His, Abcam) in blocking buffer at room temperature for 1 h with continuous shaking. After washing and drying, the blotted membrane was scanned by Sapphire Biomolecular Imager with Sapphire Capture Software V1.7.0319.0. (Thermo Scientific biosystems).

### Expression and purification of RBD

The SARS-CoV-2 RBD (residues 331-541), was cloned into the pVRC-8400 mammalian expression plasmid, with a C-terminal 6X-His-tag and an intervening HRV-3C protease cleavage site. Expression vector was transiently transfected into Expi293F GnTI^-^cells suspension culture in serum-free media (Invitrogen). Media was harvested 6 days after transfection and the secreted protein purified using Ni-NTA IMAC Sepharose 6 Fast Flow resin (GE Healthcare) followed by size exclusion chromatography (SEC) on Superdex 200 (GE Healthcare) in 10 mM, Tris pH 8.0, 150 mM NaCl (SEC buffer). For enzymatic deglycosylation of RBD was carried out by adding 1.0 mL Endo Hf (NEB) per 10 mg of RBD and incubating for 24 h at 4°C; a second round of SEC was performed to remove excess Endo Hf. Protein purity was analysed by SDS-PAGE at every step.

### IgG digestion

Fab fragments of P1D9, P2D9 and P2B4 were produced by digestion of IgG’s antibodies with immobilized Endoproteinase Lys-C (Sigma Aldrich), which was equilibrated with 25 mM Tris pH 8.5 and 1 mM EDTA for 3 h. The resulting Fabs were purified from the cleaved Fc domain by affinity chromatography using protein A. Fab purity was analysed by SDS-PAGE. All Fabs were buffer-exchanged into 20 mM Tris, 150 mM, pH 7.4 prior to cryo-EM or crystallization experiments.

### Cryo-EM data collection, processing, and structure building of P2B4

SARS-CoV-2 spike protein at a concentration of 1 mg/mL was incubated with three-fold molar excess per spike trimer of the antibody P2B4 Fab fragment for 60 min on ice in 10 mM sodium acetate pH 5.5, 150 mM NaCl, and 0.005% n-dodecyl-β-D-maltoside (DDM). Cryo-EM grids were prepared by applying 3 μL of sample to a freshly glow-discharged carbon-coated copper grid (CF 1.2/1.3 300 mesh); the sample was vitrified in liquid ethane using a Vitrobot Mark IV with a wait time of 30 s, a blot time of 3 s, and a blot force of 0. Cryo-EM data for single particle analysis were collected on a Titan Krios electron microscope operating at 300 kV, equipped with a Gatan K3-BioQuantum direct detection detector and energy filter, using the SerialEM software package [44]. Exposures were taken with a total electron fluence of 58 e^-^/Å2 fractionated over 50 frames, with a total exposure time of 2.5 s. A defocus range of −0.8 to −2.0 μm was used with a magnification of 81,000x, and a pixel size of 0.83 Å. Motion correction, CTF estimation, particle extraction, 2D classification, ab initio model generation, 3D refinements and local resolution estimation for all datasets were carried out in cryoSPARC 3.3.2 [45]. The final 3D reconstruction was obtained using non-uniform refinement with C1 symmetry. The interface between RBD and Fab was locally refined using a mask that included RBD and the variable domains of the Fab [45]. SARS-CoV-2 2P spike density was modelled using PDB entry 7DCX [46] as initial template. The initial model for P2B4 Fab variable region was obtained using the SAbPred server [47]. Automated and manual model building were iteratively performed using real space refinement in PHENIX [48] and COOT [49] respectively. Geometry validation and structure quality assessment were performed by using MOLPROBITY [50]. Map-fitting cross correlation (Fit-in-Map tool) and figures preparation were carried out using PyMOL and UCSF Chimera [51] and Chimera X [52]. A summary of the cryo-EM data collection, reconstruction and refinement statistics is shown in Table S3.

### X-ray data collection, structure solution, model building and refinement of P1D9 and P2D9

The P1D9/RBD and P2D9/RBD complexes were obtained by mixing the corresponding protein components at equimolar ratios and incubated at 4°C for O/N. The assembled complexes were further purified using the Superdex 200 Increase column and eluted with the SEC buffer. Eluted complexes were concentrated to 7.0 mg/mL for crystallization. The crystallization experiments were performed at 25°C, using the sitting-drop vapour diffusion method and diffraction quality crystals of SARS2RBD-P1D9 crystals were grown in 0.2 M ammonium sulphate, 0.1 M MES pH 6.5, 18% w/v PEG 5000MME, and SARS2RBD-P2D9 was grown complex in 2 M ammonium sulphate,0.05M MES pH 6.0 and 5 mM Magnesium acetate tetrahydrate. For data collection, the crystals were transferred to a solution containing the crystallization solution supplemented with 30% glycerol, and then flash-cooled in liquid nitrogen. Diffraction data were collected at the Advanced photon source (24ID-C and 24-IDE). Diffraction data were processed with XDS [53] and scaled using AIMLESS [54] from the CCP4 software suite (Collaborative Computational Project Number 4, 1994) [55]. Structures were solved by molecular replacement using PHASER [55] in PHENIX [48]. Iterative model building and refinement were carried out in COOT [49] and PHENIX. PISA was used to identify paratope-epitope interfaces and to calculate buried surface area [56]. A summary of the X-ray data collection, reconstruction and refinement statistics is shown in Table S4.

### Cryo-EM sample preparation of P3E6

The SARS-CoV-2 Omicron BA.1 spike ECD trimer proteins were purchased from Sino Biological Inc. (Cat: 40589-V08H26). The spike trimer (2.2 mg/mL) was mixed with P3E6 Fab (2.7 mg/mL) in 1x PBS buffer at a molar ratio of 1:1.2 (spike monomer:Fab) and incubated on ice for 40 min. 3.5 μL of spike-P3E6 complex sample was deposited to holey carbon grids (300 mesh C Flat Au R1.2/1.3) which were freshly glow discharged. The sample was incubated on the grid for 30s and plunge frozen into liquid ethane cooled by liquid nitrogen using MKIV Vitrobot (Thermo Fisher Scientific) after blotting extra samples with filter paper for 4.5 s at 4 °C and 100% humidity.

### Cryo-EM data collection, processing, and structure building of P3E6

For the complex of spike trimer and P3E6 Fab, the grids were loaded onto a Titan Krios G3i transmission electron microscope (Thermo Fisher Scientific) operated at 300 kV. Movies were collected by EPU software with a Gatan K3 Summit direct electron detector and a Bio Quantum energy filter with a 20 eV slit width. Movies were collected with a 4.5 s exposure for 40 frames, with a total dose of ∼50 e^-^/Å^2^, in counting mode at a nominal magnification of 81,000x. The defocus is set to a range of −1.0 to −2.5 μm. The 6,218 cryo-EM movies were motion corrected and dose weighted using MortionCor2 [57]. The CTF parameters estimation, particle picking and extraction, 2D classification were conducted in cryoSPARC (v 3.3.2) [45]. The initial model was constructed by picked particles with clear features and functioned as reference for further heterogeneous refinement. The results presented two states. State1 displays the binding of two Fabs, and State2 displays a closed conformation of spike trimer with no Fab bound. Further 3D classification (k = 4) without alignment in RELION 4.0 [58] separate the State1 particles into two classes: the first class contains one up RBD with P3E6 bound and two free down RBDs (1u2d), the other class present one down RBD and two up RBDs with one P3E6 connected with down and one of the up RBDs (2u1d). Particles were imported back to cryoSPARC to conduct non-uniform refinement to improve map quality. Masks were generated to cover the RBD-Fab region for the local refinement, in order to reveal the details of interactions between P3E6 Fab and RBD of Omicron spike protein. SARS-CoV-2 spike trimer with up RBD (PDB: 6Z97) [59] and Fab homology structure built by AlphaFold [60] were used as initial models to dock into cryo-EM density map. The initial models were then adjusted manually using COOT [49] and refined in COOT and PHENIX [48]. Structural analysis was conducted in UCSF Chimera [51] and UCSF ChimeraX [52]. A summary of the cryo-EM data collection, reconstruction and refinement statistics is shown in Table S5.

### Quantification and statistical analysis

Statistical analysis was performed using PRISM 8.0 or later as previously described [21]. Ordinary one-way ANOVA and multiple comparisons were used to compare group means and differences between multiple groups. Unpaired Student’s t-tests were used to compare group means between two groups only. A *P* value < 0.05 was considered significant. The number of independent replicates performed, the number of animals in each group, and the specific details of statistical tests are reported in the figure legends and the Methods section.

## Supplementary Material

Supplemental Material

## Data and materials availability statement

Cryo-EM density maps have been deposited in the Electron Microscopy Data Bank with accession codes EMD-27775 (WT 2P spike-P2B4 Fab), EMD-33892 (BA.1 RBD-P3E6 Fab local map used for model building), EMD-33893 (2u1d BA.1 spike-P3E6 Fab) and EMD-33894 (1u2d BA.1 spike-P3E6 Fab). Atomic coordinates have been deposited in the Protein Data Bank with accession codes 8DXS (WT RBD-P2B4 Fab-VH/VL), 8DWA (WT RBD-P1D9 Fab), 8DW9 (WT RBD-P2D9 Fab) and 7YKJ (BA.1 RBD-P3E6 Fab).
