# Supplementary material for: Structural insights into broadly neutralizing antibodies elicited by hybrid immunity against SARS-CoV-2
Source: Emerg Microbes Infect. 2023 Jan 3;12(1):2146538. doi: 10.1080/22221751.2022.2146538 (PMC9817130; doi:10.1080/22221751.2022.2146538)
Supplement: Supplemental Material [file TEMI_A_2146538_SM6437.pdf]

A

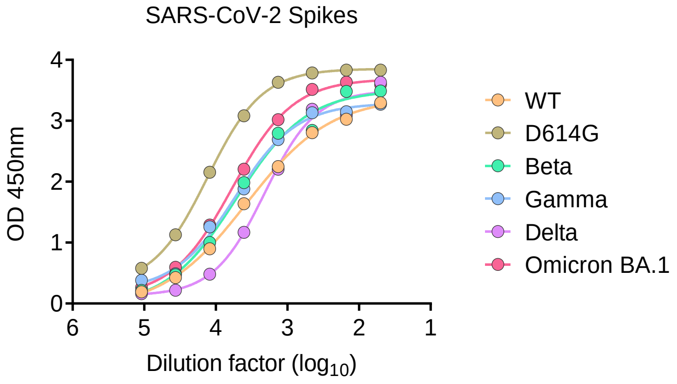

EC_{50} (Dilution factor)

**WT**	3696
**D614G**	13038
**Beta**	5104
**Gamma**	5367
**Delta**	2073
**Omicron BA.1**	5887

B

Dilution factor	WT	D614G	Alpha	Beta	Gamma	Delta	BA.1	BA.2	BA.2.12.1	BA.4/5
**IC_{50}**	11231	3869	5775	6777	4268	3274	3149	3738	2125	525.8
**IC_{90}**	593.4	282	1547	1324	289.7	115.4	411.2	222	232.1	<150
**NPI**	3.0	0.3	N/A	1.3	0.8	1.6	0.5	N/A	N/A	N/A

Figure S1. Characterization of reactive antibody responses recalled by Omicron BA.1 breakthrough infection. **(A)** The spike-specific binding activity of plasma derived from the patient Omicron-01 was measured by ELISA at serial dilutions. **(B)** The summary of IC_{50} and IC_{90} of plasma against pseudotyped SARS-CoV-2 WT and variants including Omicron sub-lineages. Neutralizing potency index (NPI) is also indicated.

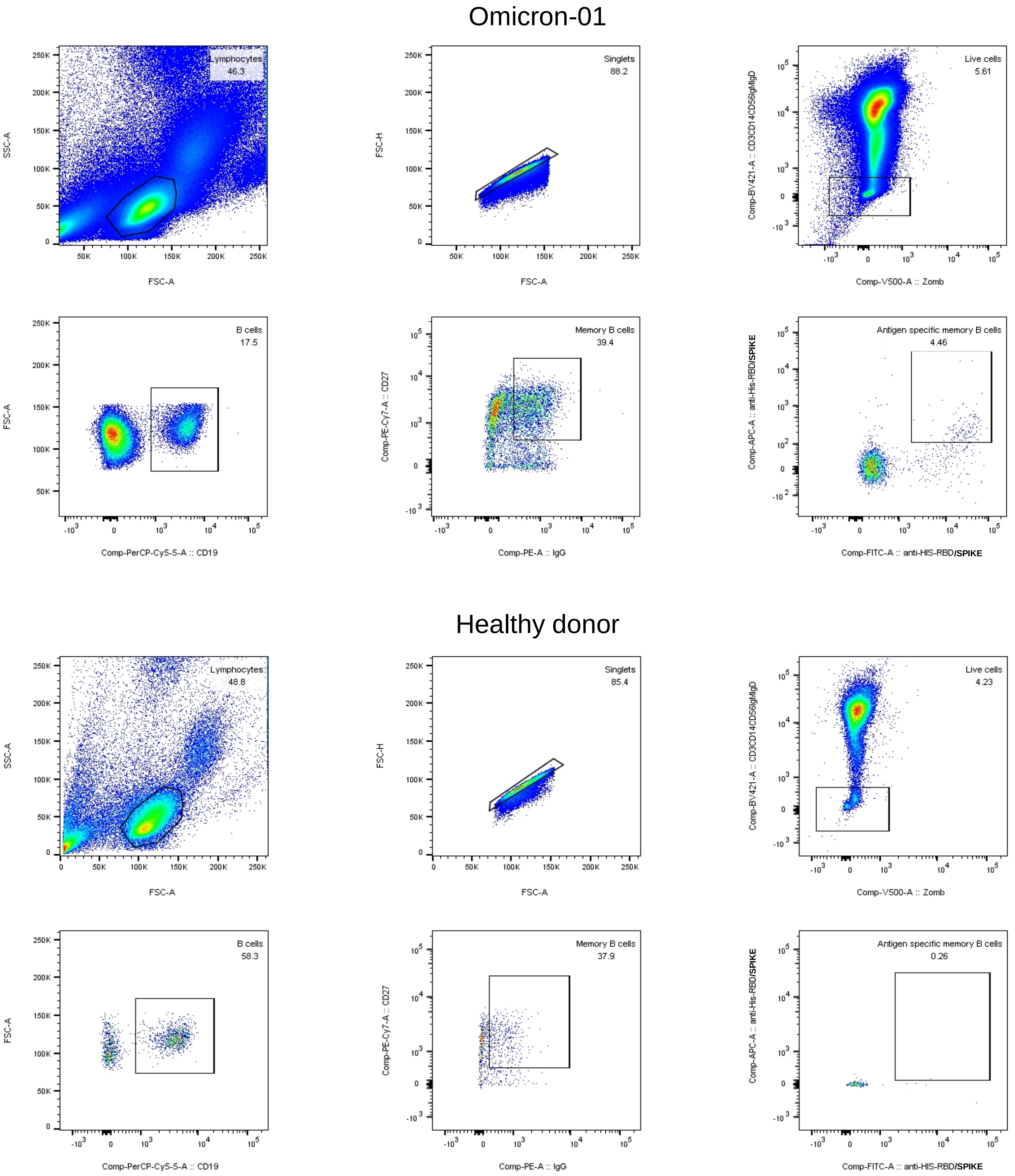

Figure S2. Isolation of antigen-specific immunoglobulin G-positive (IgG+) memory B cells. **(A)** FACS (Fluorescence-activated cell sorting) sorting of Omicron-01 PBMC into SARS-CoV-2 RBD/spike-specific $CD19^{+}CD27^{+}$ IgG^{+} subset. **(B)** FACS sorting of health donor PBMC as the negative control.

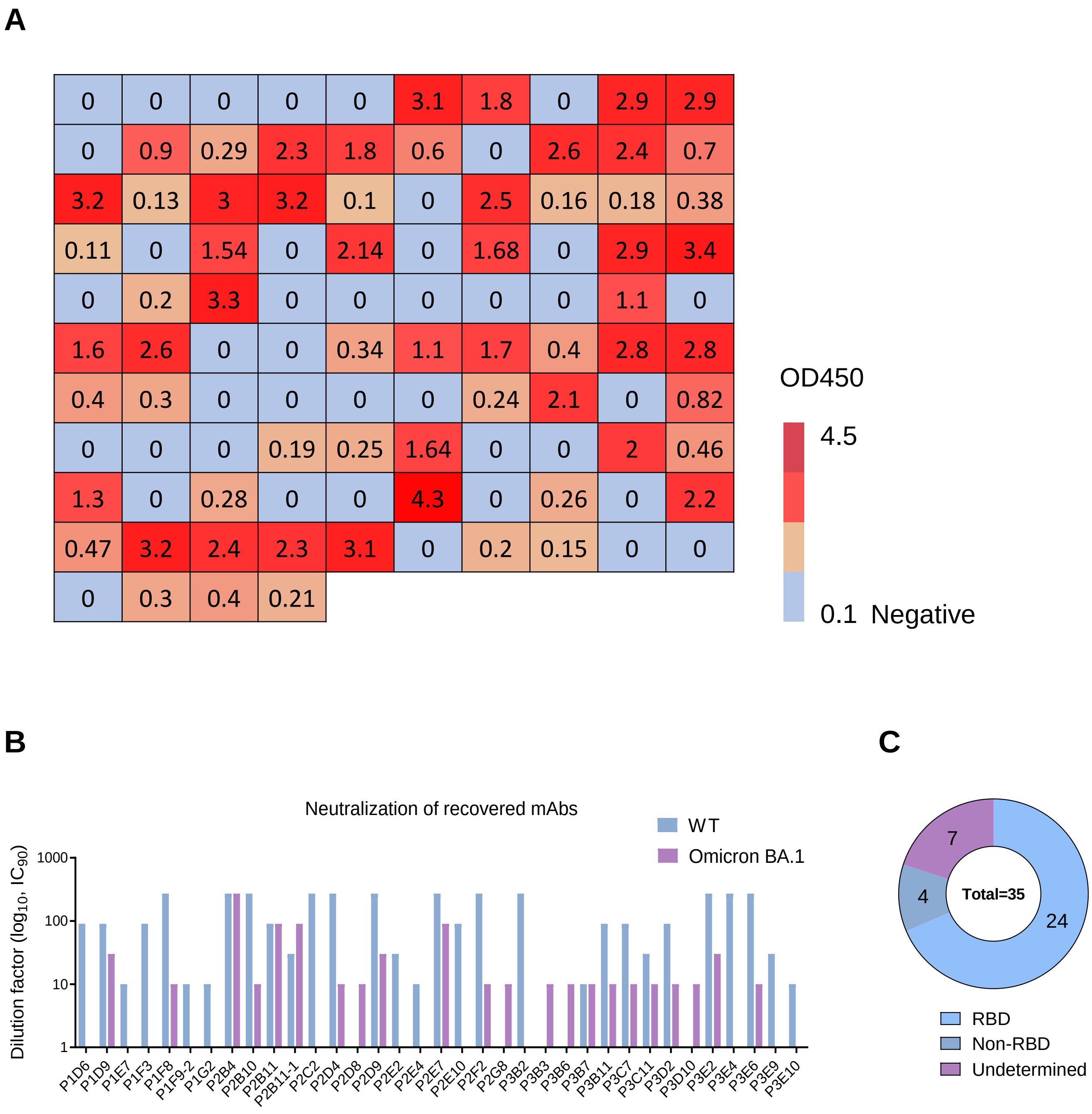

Figure S3. Binding and neutralization activity of recovered mAbs to SARS-CoV-2. **(A)** The binding activity of 104 recovered mAbs to SARS-CoV-2 WT spike was measured by ELISA. Those antibodies were successfully expressed by 293T cells and subjected to serial nonquantitative dilutions. Shown is the OD450 value of each antibody at the lowest dilution level, labelled in light blue, light orange, red and dark red from low to high value. **(B)** 35 selected mAbs with strong binding activity showed the neutralization response. Serial nonquantitative dilution assays were performed. The histogram summarizes the dilution factor of each antibody against SARS-CoV-2 WT and Omicron BA.1 with 90% neutralization. **(C)** Most of the 35 NAbs targeted WT RBD.

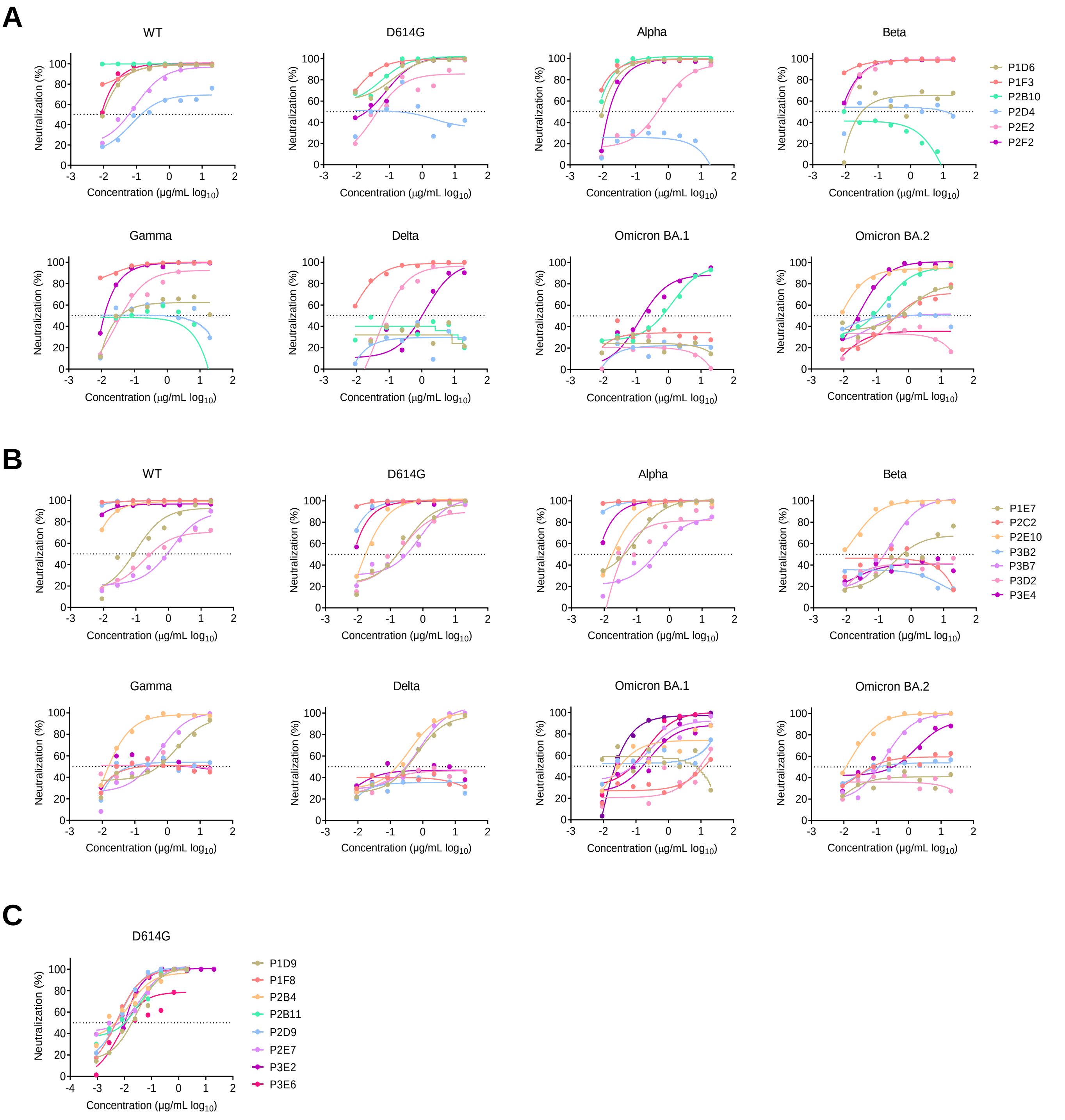

Figure S4. Quantitative neutralization measurement of newly identified NAbs against pseudotyped SARS-CoV-2 WT and variants. **(A and B)** 13 of 35 NAbs were selected based on their cross-neutralization capability and expressed by 293F cells for quantitative neutralization measurement against pseudotyped SARS-CoV-2 WT and variants. The dashed line in each graph indicates 50% neutralization. **(C)** 8 of 35 NAbs were selected based on their potent neutralization against Omicron sub-lineages and expressed by 293F cells for quantitative neutralization measurement against pseudotyped SARS-CoV-2 D614G variant.

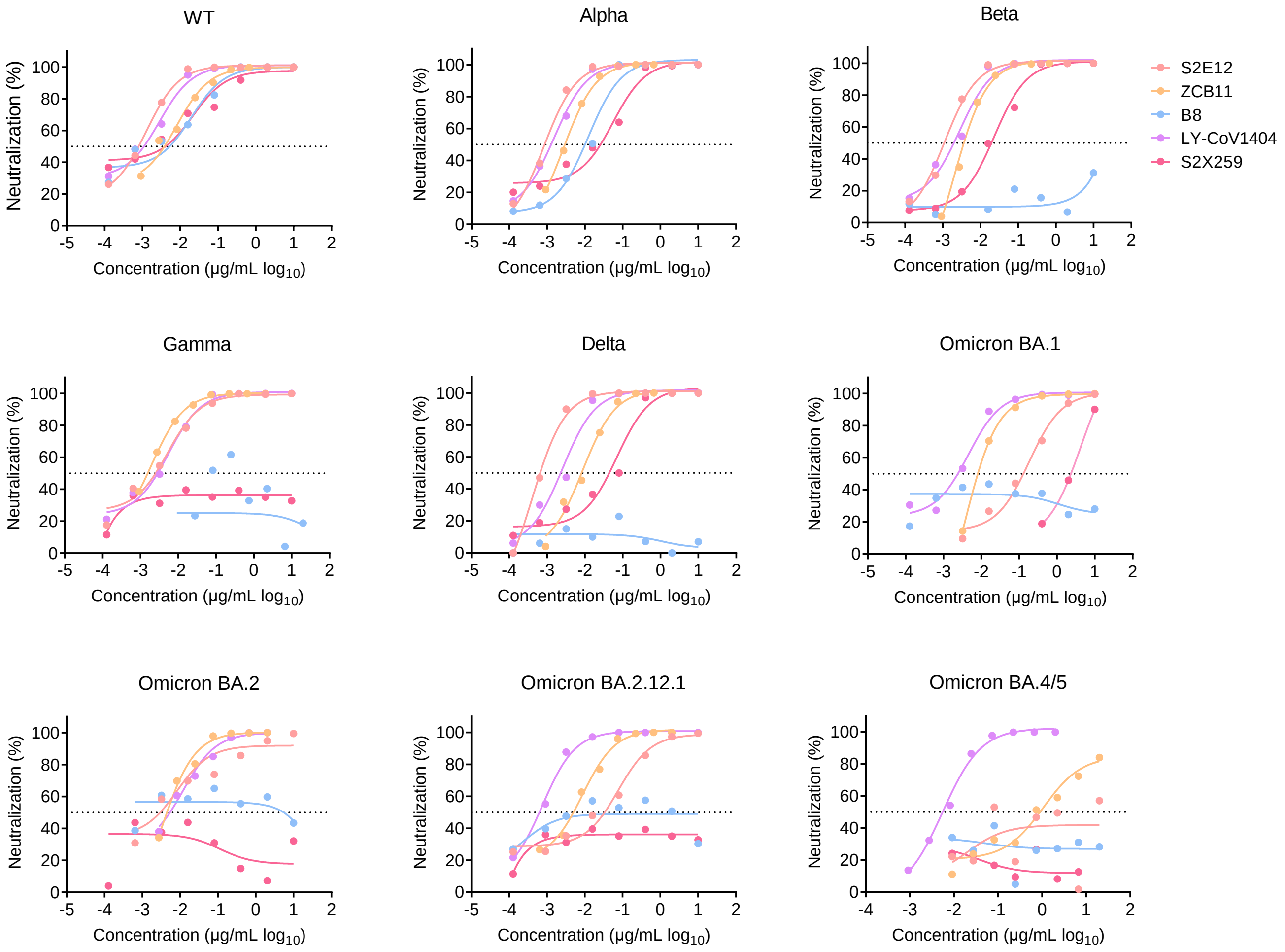

Figure S5. Quantitative neutralization measurement of published NAb controls from class I-IV (S2E12, ZCB11, B8, LY-CoV1404 and S2X259) against pseudotyped SARS-CoV-2 WT and variants.

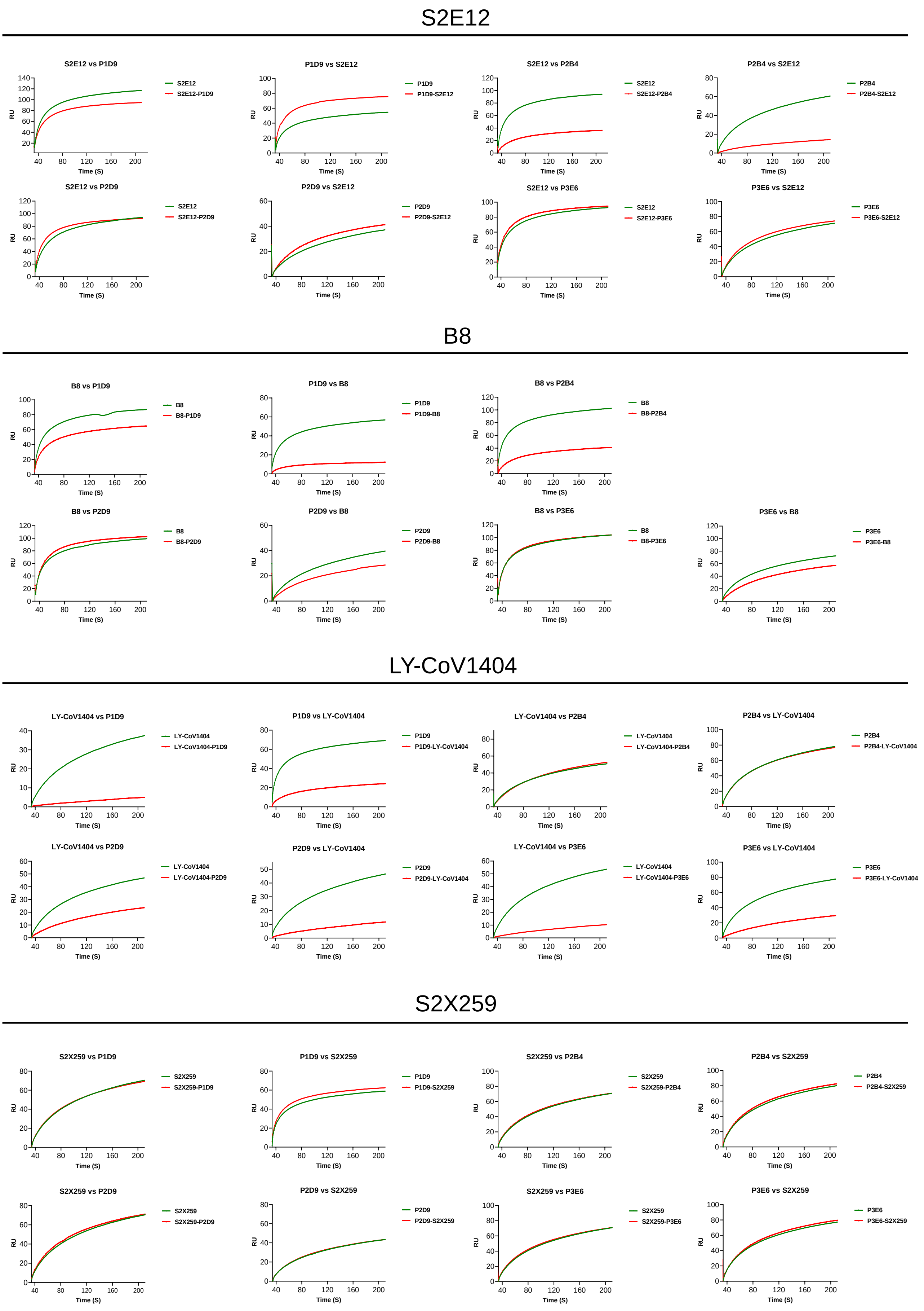

Figure S6. Competitive binding between 4 identified NAbs and published NAbs to SARS-CoV-2 WT RBD. The competition between 4 NAbs (P1D9, P2B4, P2D9 and P3E6) and published NAbs (S2E12, B8, LY-CoV1404 and S2X259) for binding to SARS-CoV-2 WT RBD was measured by surface plasmon resonance (SPR). The curves show the binding of each NAb to SARS-CoV-2 RBD without (green) or with (red) pro-incubation of competitor NAb.

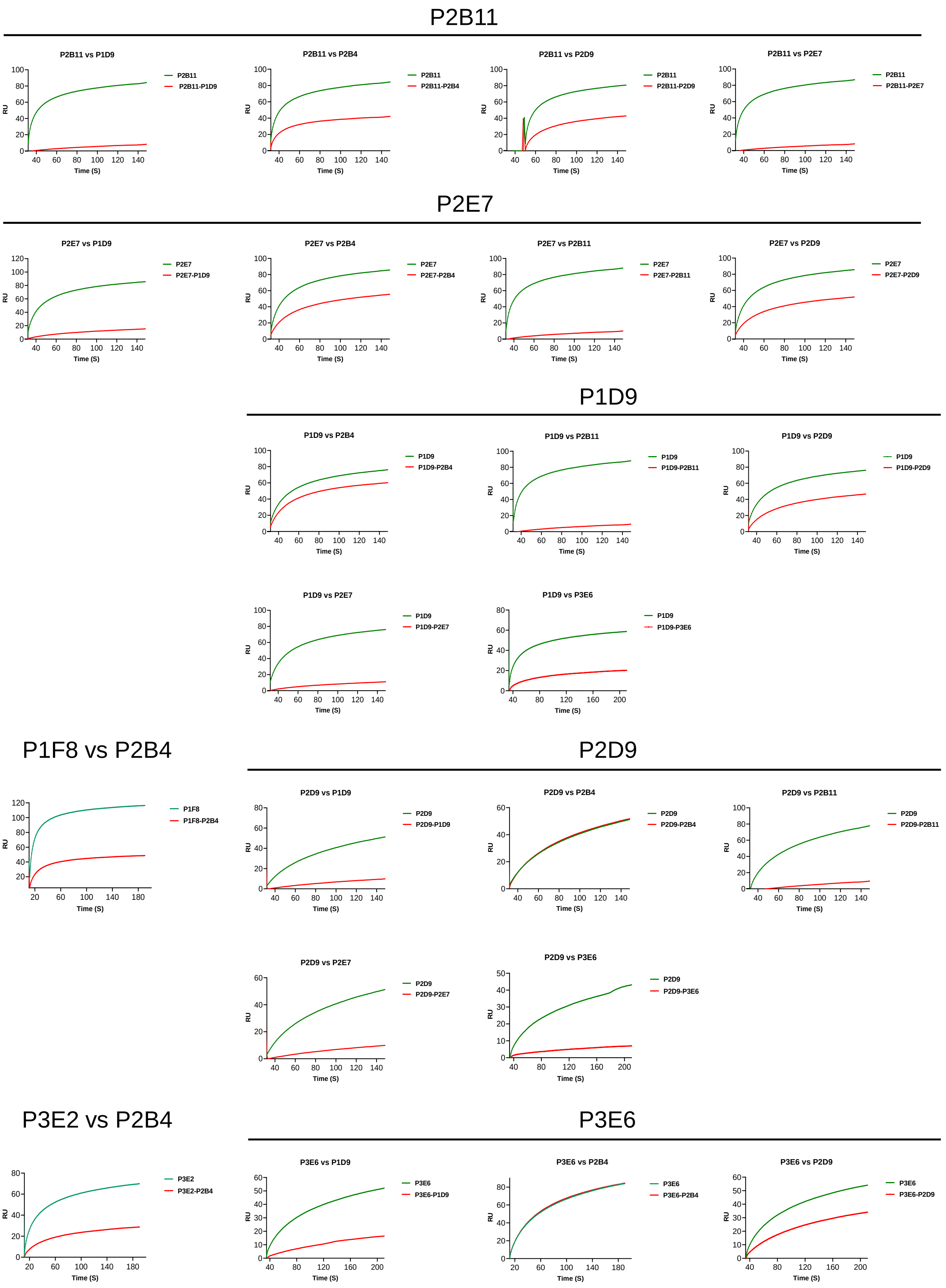

Figure S7. Competitive binding between 8 identified NAbs to SARS-CoV-2 WT RBD. The competition between 8 NAbs (P1F8, P3E2, P2B11, P2E7, P2B4, P1D9, P2D9, and P3E6) for binding to SARS-CoV-2 WT RBD was measured by surface plasmon resonance (SPR). The curves show the binding of each NAb to SARS-CoV-2 RBD without (green) or with (red) pro-incubation of competitor NAb.

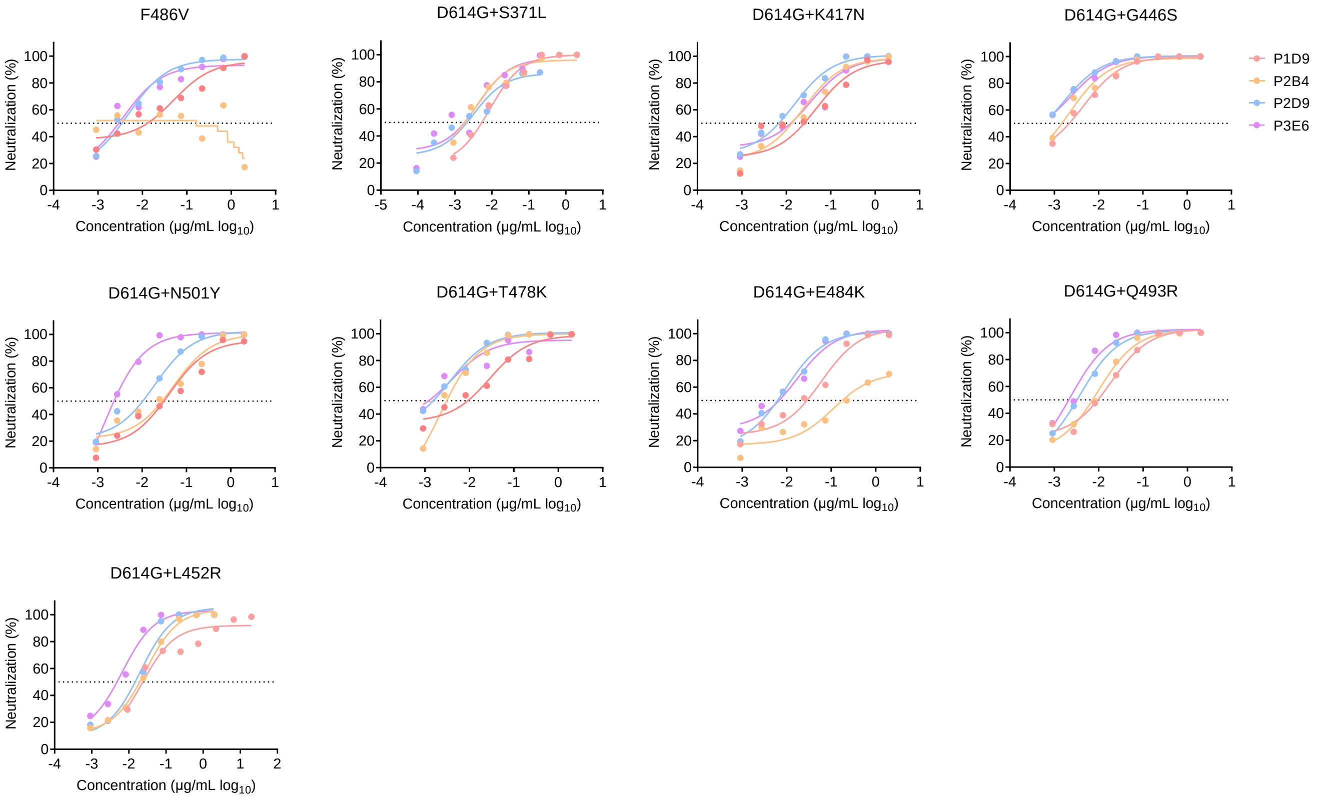

Figure S8. Identified Class II/III NAbs display diverse neutralization activity against SARS-CoV-2 WT pseudovirus with point substitutions. The neutralization activity of P2B4, P1D9, P2D9 and P3E6 against pseudotyped SARS-CoV-2 WT with F486V or D614G plus the indicated point substitutions found within the RBD. The dashed line in each graph indicates 50% neutralization.

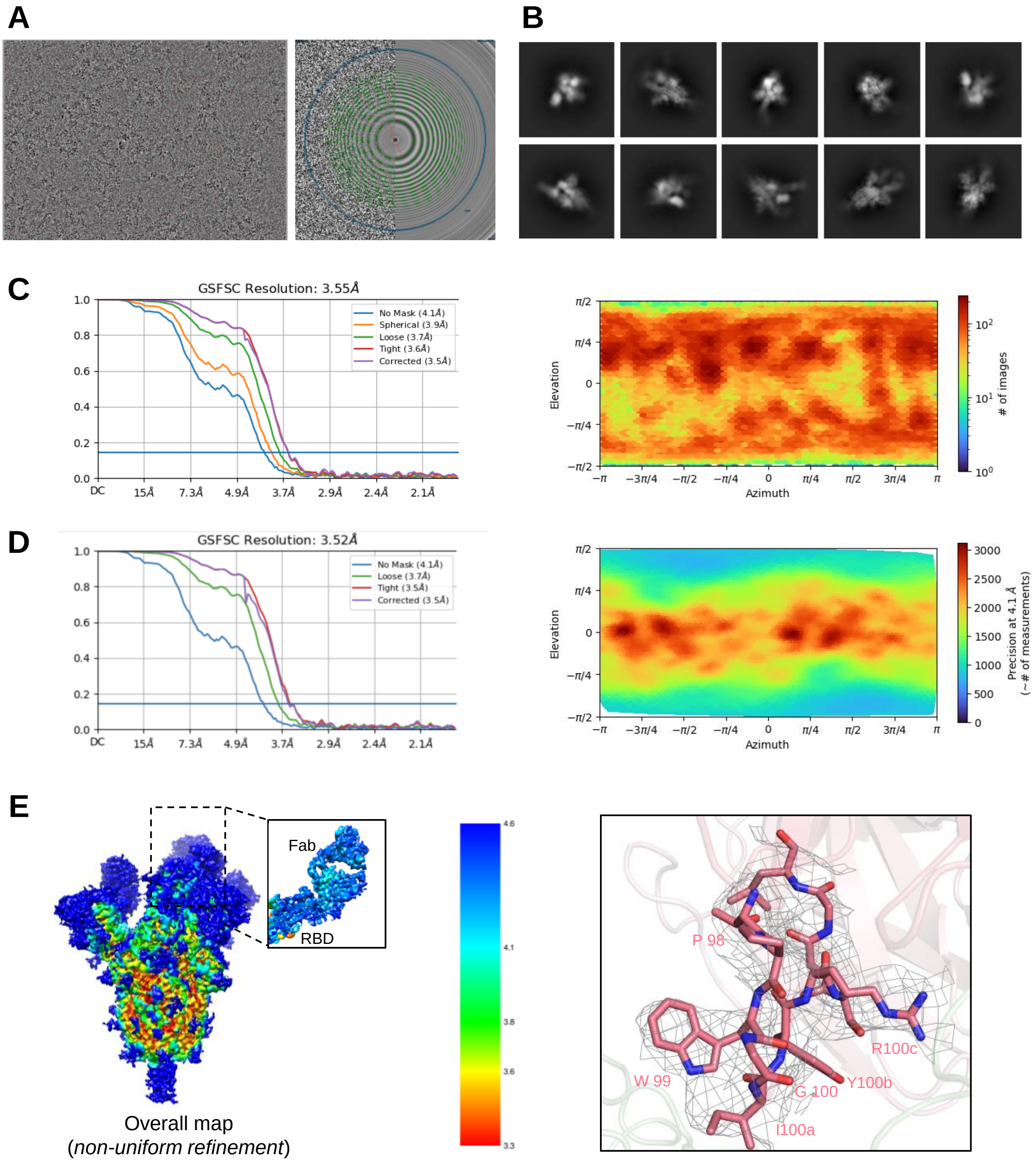

Figure S9. Cryo-EM details of P2B4 Fab in complex with SARS-CoV-2 WT 2P spike. **(A)** Representative micrograph and CTF of the micrograph are shown. Cryo-EM structure of SARS-CoV-2 WT 2P spike in complex with P2B4 Fab at 3.5 Å resolution. **(B)** Representative 2D class averages are shown. **(C)** The gold-standard Fourier shell correlation resulted in a resolution of 3.55 Å for the overall map using non-uniform refinement (left panel); the orientations of all particles used in the final refinement are shown as a heatmap (right panel); the observed preferred orientation did not preclude the generation of a high-quality map. **(D)** The gold-standard Fourier shell correlation resulted in a resolution of 3.9 Å for the masked local refinement of the RBD:P2B4 interface (left panel); the orientations of all particles used in the local refinement are shown as a heatmap (right panel). the observed preferred orientation did not preclude the generation of a high-quality map. **(E)** The local resolution of the final overall map and locally refined map are shown, generated through cryoSPARC using an FSC cutoff of 0.5. Representative density is shown for the CDR H3 loop of P2B4 contacting NTD. CDR H3 carbon atoms are colored in red, oxygen in red, nitrogen in blue; RBD is colored in pale green.

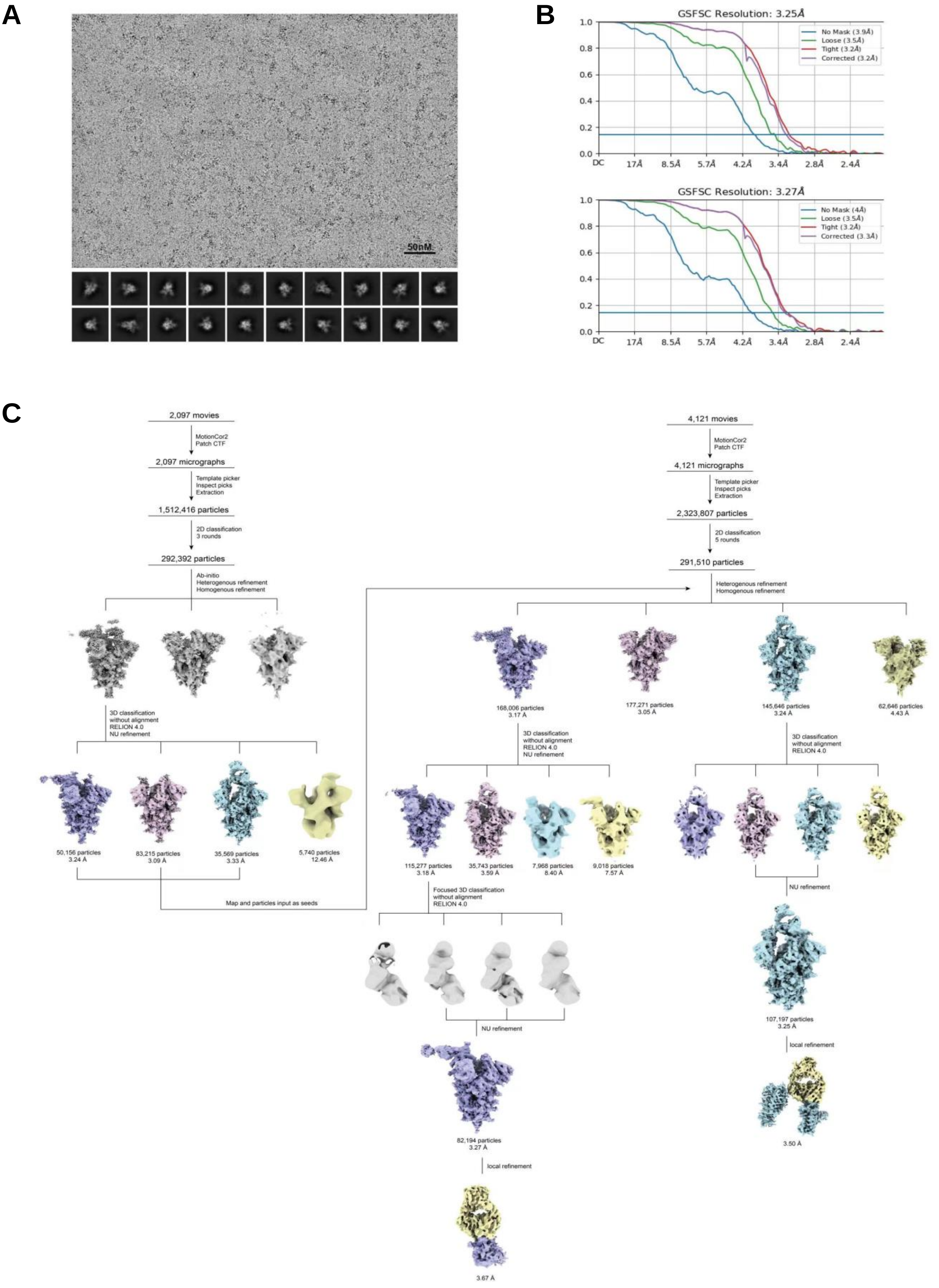

Figure S10. Cryo-EM details of P3E6 Fab in complex with SARS-CoV-2 Omicron BA.1 spike. **(A)** A representative cryo-EM micrograph of the P3E6 Fab-spike complex. Representative 2D averages from picked particles are shown below. **(B)** Gold-standard Fourier shell correlation (GFSC) curves of the density map of P3E6 Fab-spike complex with 2 up and l down RBDs (2u1d, top), as well as 1 up and 2 down RBDs (1u2d, bottom). **(C)** Data processing workflow of P3E6 Fab-spike complex.

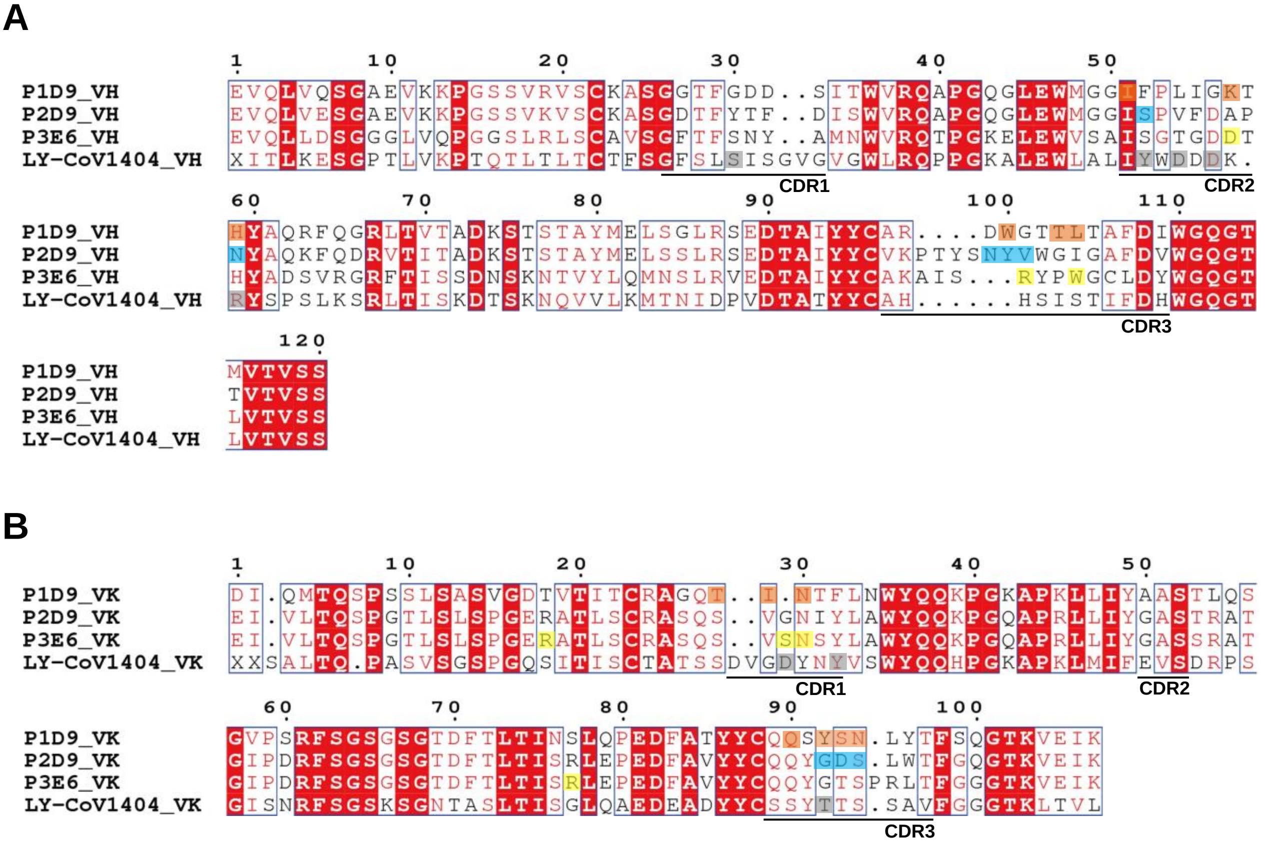

Figure S11. Sequence alignment of class III P1D9, P2D9, P3E6 and LY-CoV1404 variable regions. **(A)** Sequence alignment of VH. The residues of P1D9, P2D9, P3E6 and LY-CoV1404 VH (GenBank accession: 7MMO_A) involved in binding RBD are labelled in orange, blue, yellow and grey, respectively. CDR1/2/3 are indicated. **(B)** Sequence alignment of VL. The residues of P1D9, P2D9, P3E6 and LY-CoV1404 VL (GenBank accession: 7MMO_B) involved in binding RBD are labelled in orange, blue, yellow and grey, respectively. CDR1/2/3 are indicated.

Table S1. The WT RBD-specific binding activity of 35 NAbs was measured by ELISA

Antibody	P1D6	P1D9	P1E7	P1F3	P1F8	P1F9-2	P1G2	P2B4	P2B10
EC50 (ug/ml)	0.015	0.007	0.039	0.011	0.008	N/A	N/A	0.012	0.013
Antibody	P2B11	P2B11-1	P2C2	P2D4	P2D8	P2D9	P2E2	P2E4	P2E7
EC50 (ug/ml)	N/A	0.012	0.015	0.172	N/A	0.028	0.026	N/A	0.010
Antibody	P2E10	P2F2	P2G8	P3B2	P3B3	P3B6	P3B7	P3B11	P3C7
EC50 (ug/ml)	0.006	0.007	--	0.012	N/A	N/A	12.63	0.078	0.093
Antibody	P3C11	P3D2	P3D10	P3E2	P3E4	P3E6	P3E9	P3E10	
EC50 (ug/ml)	--	0.064	0.188	0.163	0.013	0.114	--	--	

N/A: The binding activity of 7 NAbs was not determined.

Table S2. Neutralization profiles of 21 NAbs

Antibody	Neutralization								IC$_{50}$ (ng/mL)	Symbol
	WT	D614G	Alpha	Beta	Gamma	Delta	Omicron BA.1	Omicron BA.2		
P1D6	***	***	***	X	X	X	X	X	1-10	***
**P1D9**	***	**	**	***	X	X	**	**	10-100	**
P1E7	**	*	**	X	*	X	X	X	100-1000	*
P1F3	***	***	***	***	***	***	X	X	>1000	x
**P1F8**	***	***	***	X	X	X	**	***		
**P2B4**	**	**	**	X	X	**	***	***		
P2B10	***	***	***	X	X	X	*	*		
**P2B11**	**	**	**	***	X	X	**	**		
P2C2	***	***	***	X	X	X	X	X		
P2D4	**	X	X	X	X	X	X	X		
**P2D9**	***	***	***	***	***	***	**	*		
P2E2	**	**	*	***	**	**	X	X		
**P2E7**	***	**	**	***	***	X	**	**		
P2E10	***	***	**	***	**	*	**	**		
P2F2	***	**	**	***	***	*	*	**		
P3B2	***	***	***	X	X	X	*	X		
P3B7	*	*	*	*	*	*	X	*		
P3D2	*	*	*	X	X	X	*	X		
**P3E2**	**	***	**	***	**	X	**	**		
P3E4	***	***	***	X	X	X	*	X		
**P3E6**	**	***	**	**	**	**	***	**		

Note: 8 bNAbs that neutralize Omicron variants potently are in bold for further analysis.

Table S3. Profile of WT 2P spike in complex with P2B4 Fab

SARS-CoV-2 WT 2P spike complex	P2B4 Fab
EMDB ID	27775
PDB ID	8DXS
Data Collection	
Microscope	FEI Titan Krios
Voltage (kV)	300
Electron dose (e^-/Å^2)	41.92
Detector	Gatan K3 BioQuantum
Pixel Size (Å)	1.07
Defocus Range (µm)	-0.8/-2.5
Magnification	81000
Reconstruction	
Software	cryoSPARC v3.3.2
Particles	93,569
Symmetry	C1
Box size (pix)	392
Resolution (Å) ($FSC_{0.143}$)	3.52
Refinement	
Software	UCSF Chimera, ChimeraX, Coot, Phenix
Protein residues	3791
Chimera CC	0.85
EMRinger Score	2.43
R.m.s. deviations	
Bond lengths (Å)	0.006
Bond angles (°)	1.16
Validation	
Molprobity score	1.41
Clash score	4.64
Favored rotamers (%)	2.1
Ramachandran	
Favored regions (%)	97.0
Allowed regions (%)	3.0
Disallowed regions (%)	0

Table S4. Profile of WT RBD in complex with P1D9 or P2D9 Fabs

SARS-CoV-2 WT RBD complex	P1D9 Fab	P2D9 Fab
PDB ID	8DWA	8DW9
Data Collection		
Space group	$P4_22_12$	$P\ 2_1\ 2_1\ 2_1$
Unit cell dimensions		
a,b,c (Å)	147.1 147.1 80.1	80.1 106.7 207.6
a,b,g (°)	90.0,90.0, 90.0	90.0, 90.0, 90.0
Resolution range (Å)	54.1 – 2.8 (2.9-2.8)*	94.1 - 3.95 (3.8- 3.9)*
Total reflections	394608 (40880)	33813 (3332)
Unique reflections	14941 (1449)	13088 (1296)
Completeness (%)	99.3 (99.6)	92.6 (82.4)
Redundancy	26.4 (28.2)	2.6 (2.6)
I/s(I)	15.2 (1.68)	3.3 (3.1)
Rmerge	0.018(0.25)	0.37 (0.89)
CC1/2	1.0 (0.89)	0.98 (0.65)
Refinement		
Resolution range (Å)	54.1-2.82	94.1 - 3.95
Number of complexes per asymmetric unit	1	2
Rwork/Rfree	0.21/0.29	0.31/0.37
Number of atoms		
Protein	596	1208
Ligands	27	0
Water	0	0
B-factors		
Protein	82.1	82.9
Ligands	81.5	0
Water	0	0
R.m.s. deviations		
Bond lengths (Å)	0.017	0.005
Bond angles (°)	1.79	0.84
Ramachandran statistics		
Favored (%)	96.34	90.64
Allowed (%)	3.66	9.36
Outliers (%)	0	0

Table S5. Profile of BA.1 spike in complex with P3E6 Fab

SARS-CoV-2 Omicron BA.1 spike complex	P3E6 Fab
EMDB ID	33893, 33894
PDB ID	7YKJ
Data Collection	
Microscope	Titan Krios
Voltage(kV)	300
Detector	Gatan K3 summit
Magnification	81,000x
Electron dose (e/Å^2)	50
Defocus range (μm)	-1.0~-2.5
Pixel size (Å)	1.06
Collected movies	6,218
Reconstruction	
Software	cryoSPARC v 3.3.2, RELION 4.0
Final particles	107,197
B-factors (Å^2)	-124.5
Map resolution (Å)	3.50(0.143)
Atomic modeling	
Software	UCSF Chimera, ChimeraX, Coot, Phenix
Chain	4
Residues	626
Water	0
Atoms	4811 (Hydrogens:0)
RMSD Length (Å)	0.013
RMSD Angles (Å)	1.781
Ramachandran plot (%)	
Favored	92.23
Allowed	6.63
Outliers	1.13
Rotamer outliers	2.98
C-beta outliers	0.17

Table S6. Memory B cell-derived IgGH and IgGV genes of 8 newly identified bNAbs

Antibody	IGHV gene	IGKV gene	HCDR3	HCDR3 length	Total identity
P1D9	IGHV1-69*06	IGKV1-39*01	ARDWGTTLTAFDI	13	90.8%
P1F8	IGHV3-64*01	IGKV2-28*01	ARGRRDVYTGGFDV	14	92.2%
P2B4	IGHV3-9*01	IGKV1-39*01	AKETTPWGIYRSGGLDV	17	94.3%
P2B11	IGHV1-69*06	IGKV3-20*01	ARPIQPFGDYALDV	14	94.9%
P2D9	IGHV1-69*06	IGKV3-20*01	VKPTYSNYVWGIGAFDV	17	93.1%
P2E7	IGHV4-59*01	IGKV2-28*01	ARGTTYTGFDVEWFDP	16	94.9%
P3E2	IGHV3-53*01	IGKV1-39*01	VRMMTGTDWFDP	12	94.2%
P3E6	IGHV3-23*01	IGKV3-20*01	AKAISRYPWGCLDY	14	92.9%
